# Deep learning-enhanced QSAR modeling for predicting developmental neurotoxicity based on molecular initiating events from adverse outcome pathways

**DOI:** 10.1007/s11030-025-11454-6

**Published:** 2026-01-23

**Authors:** Eufrásia de Sousa Pereira, Vinícius Alexandre Fiaia Costa, Eder Soares de Almeida Santos, Bruno Junior Neves

**Affiliations:** Laboratory of Cheminformatics, Faculty of Pharmacy, Federal University of Goiás, Goiânia, Brazil

**Keywords:** Developmental neurotoxicity, Predictive modeling, Deep learning, Cheminformatics, Explainability

## Abstract

Developmental neurotoxicity (DNT) is linked to chemical exposure that disrupts the nervous system in humans or animals. Traditional methods for assessing chemical toxicity are valuable but often time-consuming, costly, and involve significant animal use, making it impractical to meet growing demands. To address this, we developed a deep learning-enhanced QSAR modeling framework aimed at predicting binding affinities towards molecular initiating events (MIEs) and key events (KEs) within the Adverse Outcome Pathway (AOP) relevant to exposure to pesticide-contaminated cannabis. Our model was trained on data from 24,476 compounds, sourced from the ChEMBL database, and tested against 4 MIE and 6 KE tasks. The DNNs showed superior performance, with an average correlation coefficient of 0.82 ± 0.05 and a root mean square error of 0.72 ± 0.08 for the test set. To enhance interpretability, we used SHAP values to explain the model’s predictions clearly. Furthermore, ECFP4 feature contributions were mapped onto known neurotoxic compounds to highlight regions likely responsible for MIEs visually. Our results confirm that developed models accurately predict DNT and effectively identify the correct MIEs and KEs for several neurotoxicants.

**Graphical abstract:**

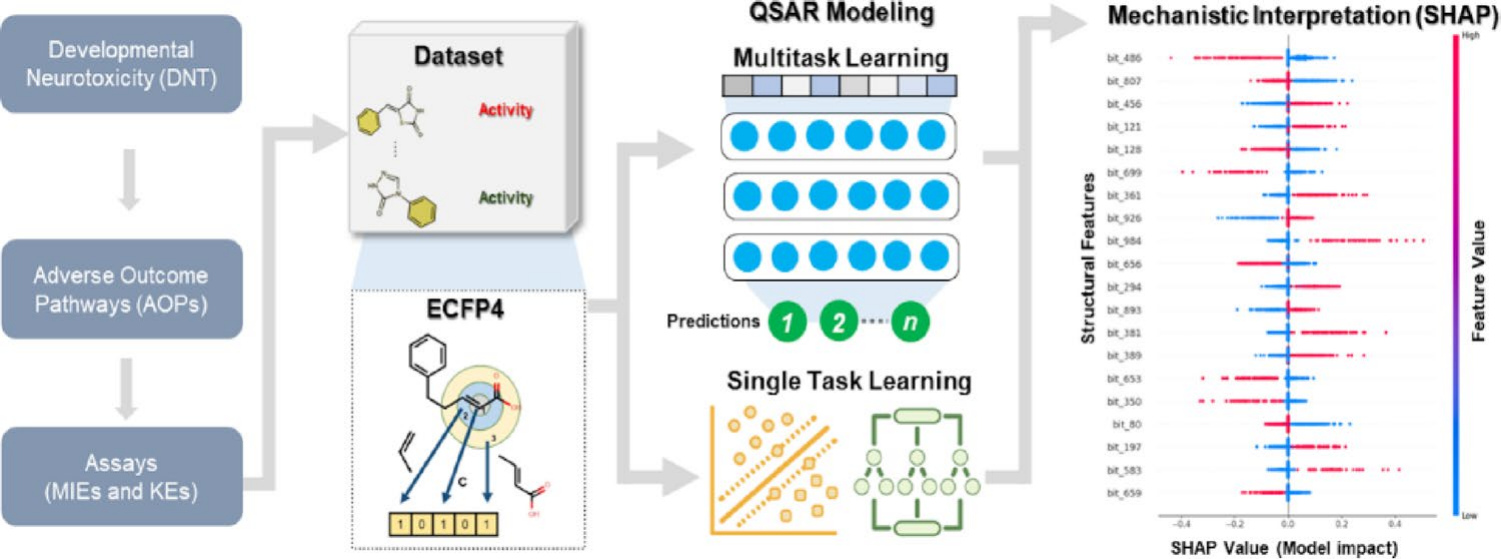

**Supplementary Information:**

The online version contains supplementary material available at 10.1007/s11030-025-11454-6.

## Introduction

The last decade has witnessed an alarming escalation in the global prevalence of neurodevelopmental disorders, highlighting the particular vulnerability of the developing brain to environmental perturbations. This growing public health challenge underscores the critical need to decipher the molecular and cellular mechanisms through which external factors disrupt neurodevelopmental trajectories, ultimately enabling more precise identification of developmental neurotoxicity hazards [1]. Strong evidence indicates that industrial chemicals widely disseminated in the environment are significant contributors to what we have called the global, silent pandemic of developmental neurotoxicity (DNT) [1]. With the commercial use of over 80,000 chemicals, a considerable number (i.e., approximately 65,000) have not undergone thorough assessment [2]. This issue becomes increasingly pressing in the context of the rising global incidence of neurodevelopmental disorders. This amplifies public concern regarding the insufficiency of DNT data for a broad spectrum of chemicals. This situation highlights a substantial gap in our knowledge and underscores the urgent need for comprehensive toxicological assessments [1, 3].

The data scarcity is partly due to DNT evaluations not being obligatory unless specific triggers are met, such as evidence of developmental toxicity affecting the nervous system or neurotoxicity in rodents [4]. Furthermore, conventional DNT investigations, which the US Environmental Protection Agency estimates to be resource-intensive (costing approximately $771,600) and predominantly reliant on using many animals for an extended period, are impractical for the resource-expensive. This methodology’s approach demands and time requirements render it unsuitable for the high-throughput evaluation necessary in toxicological research [5]. Additionally, the efficacy of rodent models to accurately predict human DNT outcomes has faced criticism due to significant inter-species differences [6]. Consequently, there is a growing call for more efficient, cost-effective, and reliable methods for DNT assessment [4, 5].

Aligning with contemporary research trends, many in vitro alternative methods have been developed to study human-specific DNT effects in a high throughput manner. Meanwhile, the expansion of our understanding of DNT molecular mechanisms aids in identifying Adverse Outcome Pathways (AOPs) related to DNT [7, 8]. An AOP outlines the progression from a molecular initiating event (MIE), such as a chemical interacting with a molecular target, to an adverse outcome (AO), via a sequence of key events (KEs) [9]. This framework allows for categorizing chemicals into different levels of concern based on the activation and extent of MIEs. Chemicals activating similar MIEs/KEs concerning known toxicants are likelier to exhibit toxicity. Consequently, this approach simplifies the identification of potential DNT effects of chemicals, bypassing the need for extensive validation using in vivo approaches [10, 11].

The elucidation of the AOP for developmental neurotoxicity represents a significant advancement in our approach to understanding and mitigating the risks of neurotoxic chemicals. For instance, the AOP framework provided by Leung et al. [12], which focuses on prenatal exposure to cannabis contaminated with organophosphate pesticides, highlights the complex pathways through which neurodevelopmental processes can be disrupted. Bridging the understanding of this AOP for developmental neurotoxicity with computational toxicology offers a promising direction in assessing chemical risks. Several authors have recently highlighted a possible synergism between the AOP concept and computational toxicology [13–17]. In this context, Quantitative Structure-Activity Relationship (QSAR) models are powerful tools for predicting the affinity of untested chemicals MIEs and KEs from their chemical structures, offering a rapid and resource-efficient means to assess potential neurotoxicity [18].

In recent years, the prediction power of QSAR models has seen substantial enhancement through the integration of deep learning (DL) techniques [19–22]. DL is an advanced machine learning technique that utilizes artificial neural networks with multiple layers or deep neural networks (DNNs) to model complex relationships between experimental properties and chemical features. DNNs operate by passing input data through successive layers, where each layer performs increasingly complex transformations on the data, enabling the extraction of intricate patterns and relationships [23]. DL models offer improved accuracy and predictive performance over traditional machine learning approaches due to their ability to process and learn from vast datasets [23, 24]. This advancement has been pivotal in streamlining the identifying potential neurotoxic agents, thereby significantly improving the efficiency and reliability of computational toxicology assessments.

In this study, we developed an integrated deep learning-enhanced QSAR modeling framework aimed at predicting the binding affinities towards MIEs and KEs, as outlined in the AOP framework provided by Leung et al., which are linked to developmental neurotoxicity (DNT) potential [12]. Utilizing bioassays that focus on these MIEs and KEs as dependent variables, we applied diverse machine learning techniques to evaluate their prediction power. Therefore, this framework could serve as an effective first tier of an Integrated Approaches to Testing and Assessment (IATA), enabling the swift evaluation of untested chemicals. It delivers insights into possible MIEs and KEs, helping toxicologists prioritize chemicals for additional and better-targeted in vitro and in vivo investigations.

## Methods

The methodological workflow is presented in detail in Supplementary Fig. S2.

### Data collection and curation

MIEs and KEs linked to neurotoxicity were identified from the AOP networks [25] published by Leung et al. [12]. Subsequently, bioassays half maximal inhibitory concentration (IC_50_) and inhibitory constant (Ki) data targeting the associated MIEs and KEs were collected from the ChEMBL database [26, 27]. The list of the bioassays related to MIEs and KEs for neurotoxicity is presented in Table 1. Then, all chemical entries in simplified molecular-input line-entry system (SMILES) format and corresponding bioactivity data were carefully curated according to the protocols proposed by Fourches et al. [28–30]. Briefly, specific chemotypes such as nitro groups and aromatic rings were normalized, whereas duplicates, salts, mixtures, polymers, and organometallic compounds were removed. Finally, the IC_50_ and Ki values were transformed into their negative logarithmic (–Log) scale pIC_50_ and pKi to ensure numerical stability during the development of models. Subsequently, we conducted the analysis and removal of duplicates in the following manner: (i) duplicates were examined visually; (ii) in cases where duplicates exhibited discordant bioactivities, specifically pIC_50_ values diverging by > 0.3 log units, both entries were excluded; and (iii) if the reported pIC_50_ values differed by ≤ 0.3 log units, an average of the pIC_50_ values was calculated, and a single entry was maintained in the dataset.

**Table 1:** List of modeled endpoints from the chembl database: associated MIE or KEs, species, and chemical entry counts before and after data curation

MIE or KE	ChEMBL ID	Target		Species	Number of entries	
					Before data curation	After data curation
MIE1	CHEMBL218	CB1	IC_50_	*Homo sapiens*	3810	2579
MIE1	CHEMBL253	CB2	IC_50_	*Homo sapiens*	4105	3419
MIE1	CHEMBL4191	MAGL	IC_50_	*Homo sapiens*	1225	661
MIE1	CHEMBL220	AchE	IC_50_	*Homo sapiens*	8832	4693
KE1	CHEMBL2056	D_1_	Ki	*Homo sapiens*	2683	981
KE1	CHEMBL217	D_2_	Ki	*Homo sapiens*	12,011	6133
KE1	CHEMBL2093864	α_2_	Ki	*Rattus norvegicus*	1835	452
KE1	CHEMBL211	M_2_	Ki	*Homo sapiens*	2278	1183
KE1	CHEMBL2094124	NMDA	IC_50_	*Homo sapiens*	1580	1390
KE2	CHEMBL4079	GRK2	IC_50_	*Homo sapiens*	315	296

MIE1, disruption of the endocannabinoid system by serine hydrolases; KE1, alteration of post-synaptic retrograde signaling; KE2, alteration of receptor tyrosine kinase signaling; CB1, cannabinoid receptor 1; CB2, cannabinoid receptor 2; MAGL, Monoacylglycerol lipase; AchE, acetylcholinesterase; D_1_, dopamine 1 receptor; D_2_, dopamine 2 receptor; α_2_, adrenergic receptor alpha-2; M_2_, muscarinic acetylcholine receptor M2; NMDA, N-methyl-D-aspartate glutamate receptor; GRK2, G-protein coupled receptor kinase 2

### Chemical space analysis

Datasets that underwent curation were subjected to analysis through the creation of a similarity map, which was produced utilizing the OSIRIS v.05.02.01 [31]. This map employs a Rubberbanding Forcefield technique to represent the similarity (vertices) among compounds (nodes). The methodology encompasses several phases: (i) initial random placement of all compounds within a two-dimensional space; (ii) computation of a similarity matrix for all compounds based on Tanimoto coefficients (Tc) and FragFP descriptors; (iii) identification of the closest neighbors (Tc > 0.8) for each compound; and (iv) gradual adjustment of the compounds’ positions to ensure that molecules with higher similarity are situated in proximity to one another [31].

### QSAR modeling protocol

To ensure the reproducibility of our study, all the codes and datasets are available on GitHub: https://github.com/LCi-UFG/Multitask-Learning.

### Split of datasets

The curated datasets were imported into Python and subjected to two splitting strategies to prepare them for model training and evaluation. Initially, the datasets were randomly divided into a training set, validation set, and test set at a ratio of 8:1:1 [32]. The training set is utilized to build the model, the validation set for hyper-parameter optimization, and the test set for evaluating the model’s performance. Besides the random splitting, a scaffold splitting approach available on Chemprop v.1.5.2 [32], was employed. This method ensures that the training, validation, and test sets do not share chemical structures with similar Bemis-Murcko scaffolds, thereby preventing model overfitting on particular structural motifs. The statistical performances of the models were reported as the mean results from five splitting runs, providing a more reliable estimate of model efficacy.

Additionally, to complement this approach and enhance the robustness of our ML models, we implemented a 5-fold cross-validation technique. This method randomly splits each dataset into five folds, ensuring each fold serves as a test set. In contrast, the others collectively form the training set, thus allowing for a comprehensive evaluation of model performance across different subsets of the data. For performance comparison purposes, we ensured the consistency of all datasets (training set and test set) across both deep learning (DL) and ML methods.

### Molecular fingerprints calculation

Before building the models, the standardized SMILES strings were encoded into extended connectivity fingerprint with diameter 4 (ECFP4) and bit vector of 1048 bits using RDKit package v.2020.03.1 [33] in Python programming language [34].

### Single-task learning protocol

Single-task (ST)-QSAR models, targeting specific curated entities associated with MIEs and KEs for neurotoxicity, were constructed using ECFP4 fingerprints as input features. These models were developed employing various methodologies, including Random Forest (RF) [35], Light Gradient Boosting Machine (LightGBM) [36], and Deep Neural Network (DNN) [37]. Models developed with RF and algorithm were implemented in Scikit-learn v.0.24.2, while LightGBM models were developed using the LightGBM package. These machine learning models were built employing a 5-fold cross-validation approach, and their hyperparameters were optimized through a Bayesian methodology, implemented in Scikit-Optimize version 0.7.4.The DNN models were developed using CUDA v.10.1 and TensorFlow framework v.2.9, implementing an 8:1:1 split for training, validation, and testing phases to ensure rigorous model evaluation. Given the complexity and the significant computational demands of the explored architectures, a random search strategy, informed by prior experience, was adopted for hyperparameter optimization, focusing on parameters such as dropout rates (0.1–0.6), learning rates (1 × 10^− 1^ – 1 × 10^− 8^), batch sizes (24–96), activation functions (Gelu, Elu, Selu, Tanh, ReLU, and Linear), regularization (L1 and L2), and optimization functions (Adam, Nadam, SGD, and RMSProp). The early-stopping approach is used to avoid overfitting and save computational resources. A maximum epoch was set as 2000. The training process was terminated early if the performance metric had not improved in 10 epochs on the validation set.

### Multitask learning protocol

**Fig. 1:**
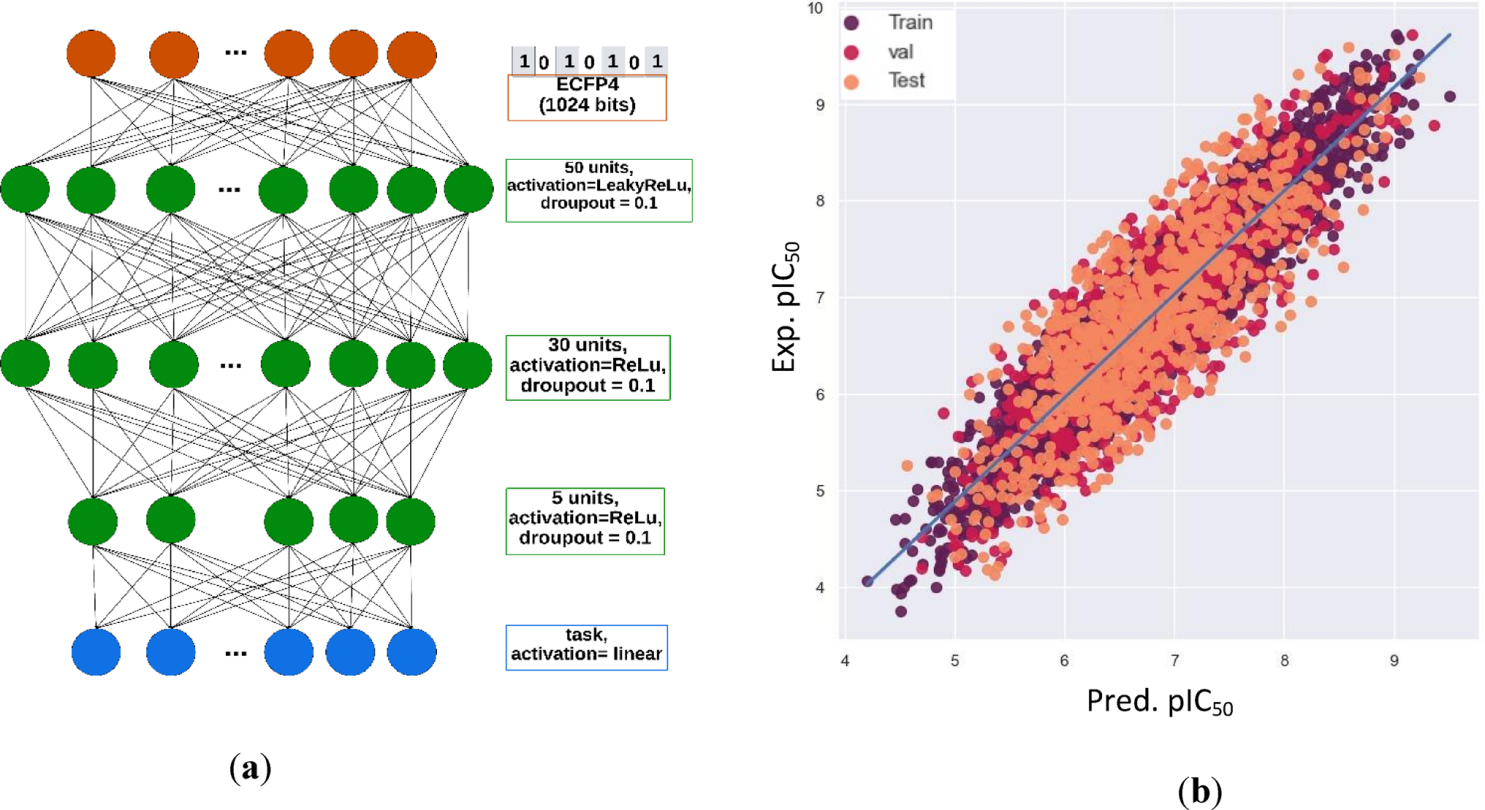
The MT-DNN architecture of multitask regression model (**a**) and scatter plot of predicted vs. experimental pIC_50_ values (**b**)

We extended the methodology applied in the ST-QSAR framework to accommodate the simultaneous learning of multiple related tasks. The multitask learning models were developed using a DNN architecture with an 8:1:1 split for training, validation, and testing to ensure reproducibility and consistent evaluation across all tasks. We employed a similar random search strategy for hyperparameter optimization to address the complexity and computational requirements inherent to MT-DNN. However, the key distinction in our multitask approach lies in integrating a multitask matrix to model shared and task-specific representations, enhancing the models’ ability to leverage commonalities and differences across tasks. This methodology underscores our commitment to developing robust models that can efficiently process and learn from multiple related tasks simultaneously, aiming for improved generalization and performance. The MT-DNN architectures were optimized using a hard parameter-sharing approach to address the overfitting issue. The same hyperparameter ranges and optimization strategy explored in single-task learning (STL) were applied. This includes dropout rates, learning rates, batch sizes, activation functions, regularization techniques, and optimization functions. The optimization results indicated that the optimal network architecture consists of a batch size of 48 samples and the Nadam optimization function with a learning rate of 1^–3^. The parameters in the network were updated using a masked mean-square error (MSE) loss function. The remaining parameters of the MT-DNN architecture are presented in Fig. 1a. Scatter plots of predicted vs. experimental pIC_50_ values for acetylcholinesterase, demonstrating a high correlation between predicted and experimental pIC_50_ values, are shown in Fig. 1b. The scatter plots for the remaining targets are provided in Fig. S3 of the Supplementary Material.

### Model evaluation

The predictive performance of the models was evaluated using the Pearson correlation coefficient (*r*), root-mean-square error (RMSE), and mean absolute error (MAE), as shown in Eqs. (1), (2), and (3), respectively:

1 $$\:r=\:\frac{\sum\:({\widehat{Y}}_{i}\:-\:\stackrel{-}{\widehat{Y}})({Y}_{i}\:-\:\stackrel{-}{Y})}{\sqrt{\sum\:({\widehat{Y}}_{i}\:-\:\stackrel{-}{\widehat{Y}}{)}^{2}\sum\:({Y}_{i}\:-\:\stackrel{-}{Y}{)}^{2}}}$$

2 $$\:RMSE=\:\sqrt{\frac{1}{n}{\sum\:}_{i}^{n}({\widehat{Y}}_{i}-\:{Y}_{i}{)}^{2}}$$

3 $$\:MAE=\:\frac{1}{n}\sum\:_{i=1}^{n}\left|{\widehat{Y}}_{i}-\:{Y}_{i}\right|\:\:\:\:$$

where *n* is the number of compounds; $\:{Y}_{i}$ and $\:{\widehat{Y}}_{i}$ are the observed and predicted values of each particular compound;$\:\:\stackrel{-}{Y}$ and $\:\stackrel{-}{\widehat{Y}}$ represent the averages of the observed and predicted values.

### Model explainability

Feature contributions were assessed following the SHapley Additive exPlanations (SHAP) values as recently reported by Rodríguez-Perez and Bajorath [38]. Herein, this algorithm was adapted for the MT-DNN regression model using the model-independent kernel SHAP approach. Kernel SHAP calculations were carried out for each target (task) of the model to interpret predictions for individual tasks. Then, multiple SHAP visualizations were combined for the comparative interpretation of activity predictions against different targets.

## Results and discussion

### Data analysis

A dataset consisting of 21,787 compounds evaluated across 10 assays was curated to develop QSAR models. The dataset covers a wide range of biological pIC_50_ or pKi values, with the lowest potency value of 3.17 (or 666 µM) and the highest potency at a pIC_50_ value of 10.0 (or 0.0001 µM). This broad range indicates that the data likely include both very active and less active compounds, which is beneficial for developing a model that can distinguish between different levels of biological activity.

**Fig. 2:**
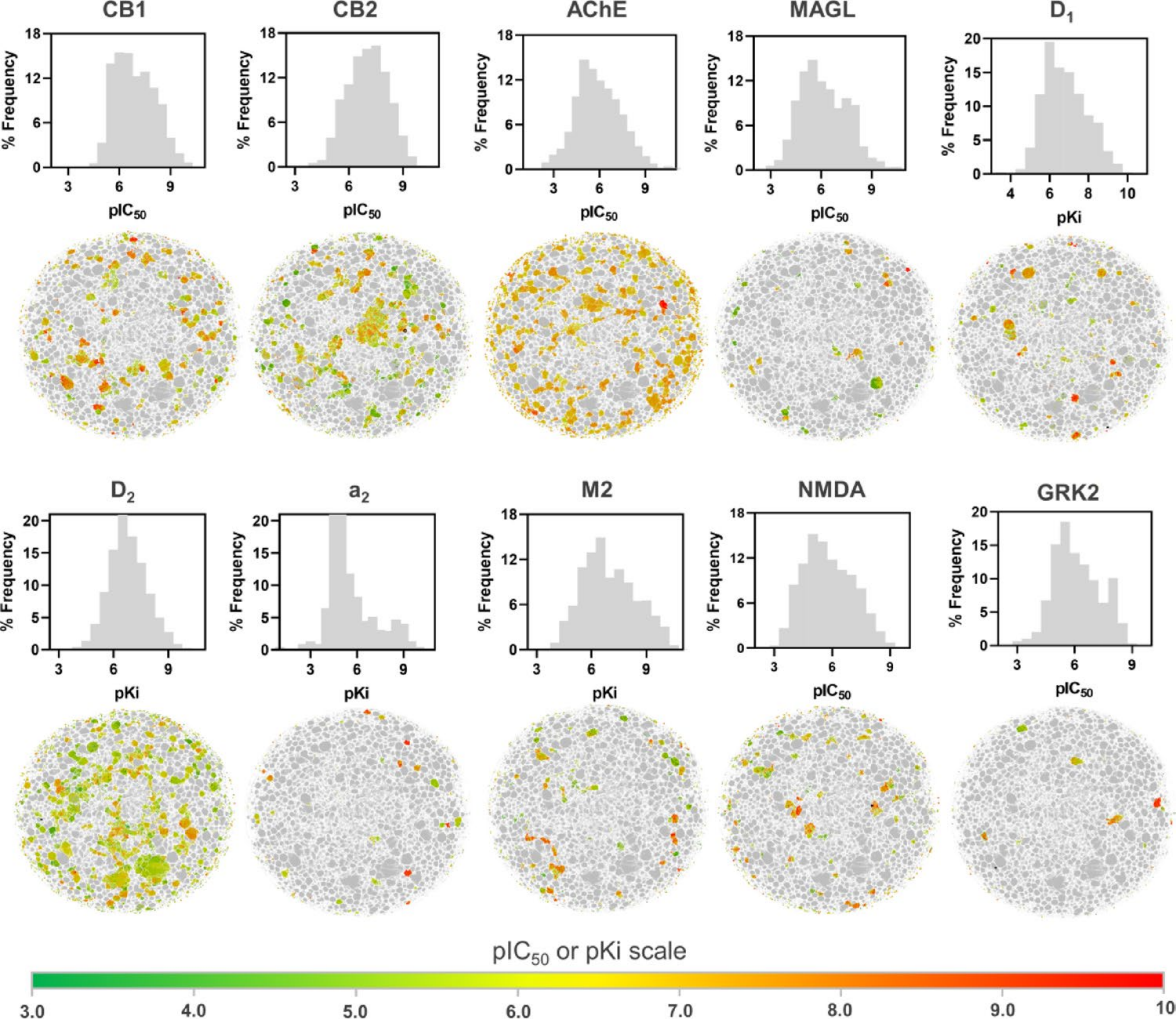
Visualization of bioactivity data distribution and structural diversity across curated chemical datasets in the entire modeling space

A graphical representation (Fig. 2) illustrates the distribution of pIC_50_ or pKi values for the 10 tasks, covering a range of potencies from 666 µM (pIC_50_ = 3.17) to 0.0001 µM (pIC_50_ = 10.0). This figure also underscores the diversity in potencies, further exemplified by the pIC_50_ values’ logarithmic span of 5 to 7 units, establishing a robust and consistent dataset for QSAR modeling.

The analysis of Pearson correlation coefficients between each task pair involved detailed calculations, revealing moderate to negative correlations among the pIC_50_ values for the selected tasks, as depicted in Fig. 2. This observation suggests the high specificity of bioassays and hints at the potential impact of interlaboratory variability, such as experimental error, on the compiled data.

### Split data

**Fig. 3:**
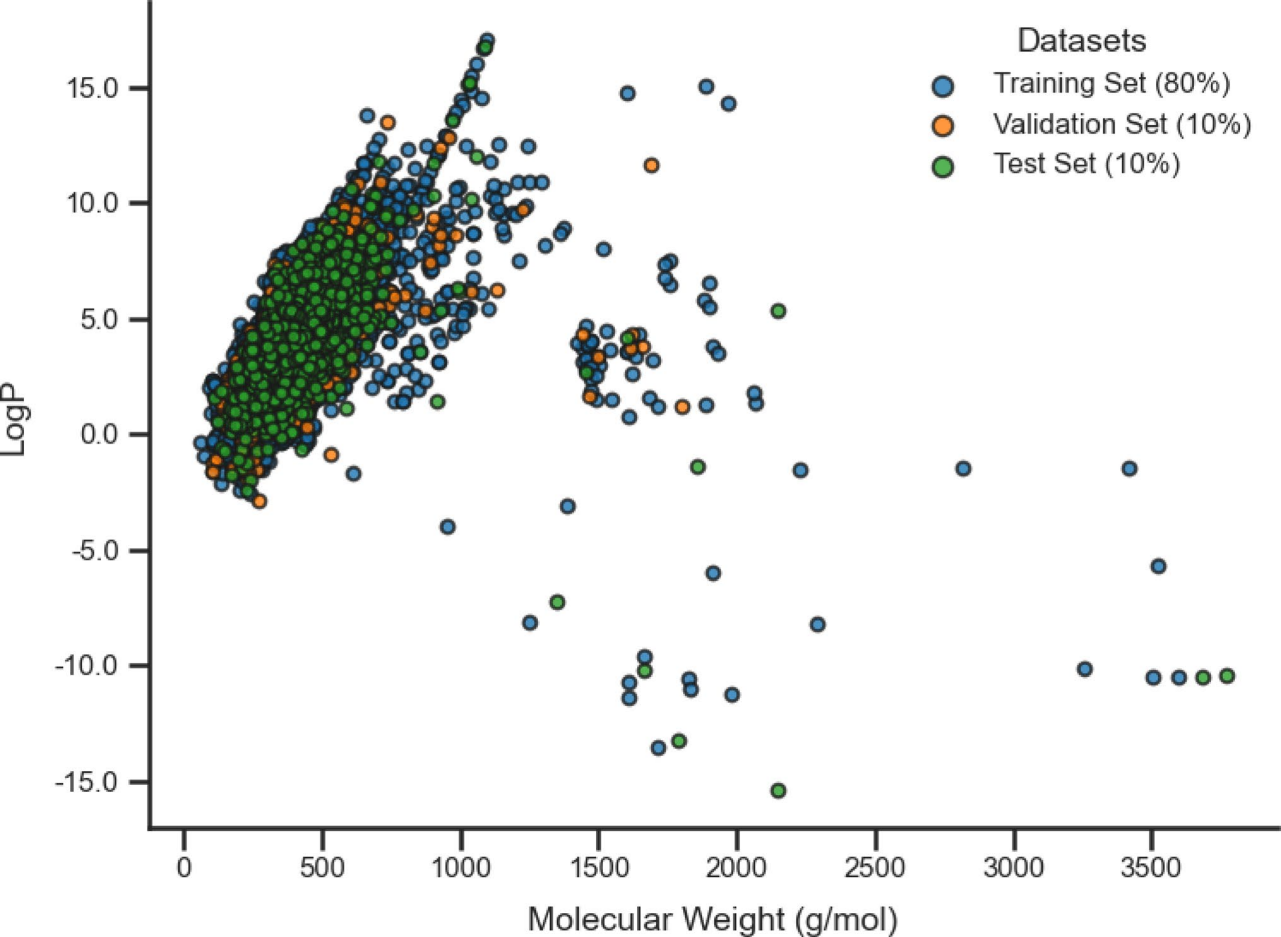
Distribution of LogP versus Molecular Weight across the training, validation, and test sets

An approach to verify whether the model is learning appropriately and making predictions confidently while avoiding the risk of overfitting is to analyze the distribution of LogP versus Molecular Weight, as shown in Fig. 3. This plot allows for the evaluation of the dispersion of samples across the training, validation, and test sets, providing insights into the diversity of the dataset. A balanced distribution suggests that the model has been exposed to a sufficient variety of compounds, which supports learning general patterns and enhances its ability to generalize to new data. When the samples are well-distributed, the model tends to be more robust and less prone to excessive fitting to the training data.

Figure 3 illustrates the LogP and Molecular Weight values distribution across the dataset. Most samples are clustered around a LogP value near zero, while the Molecular Weight values exhibit a wider spread, indicating a diverse representation of compounds with varying physicochemical properties. Although the validation and test sets are less densely populated than the training set, they still capture a broad spectrum of chemical features, which is crucial for ensuring the model’s ability to generalize to unseen data. This diversity in the dataset reflects the model’s exposure to a comprehensive range of molecular characteristics, fostering its ability to make reliable and accurate predictions while minimizing the risk of overfitting the training data.

### Model performance

**Fig. 4:**
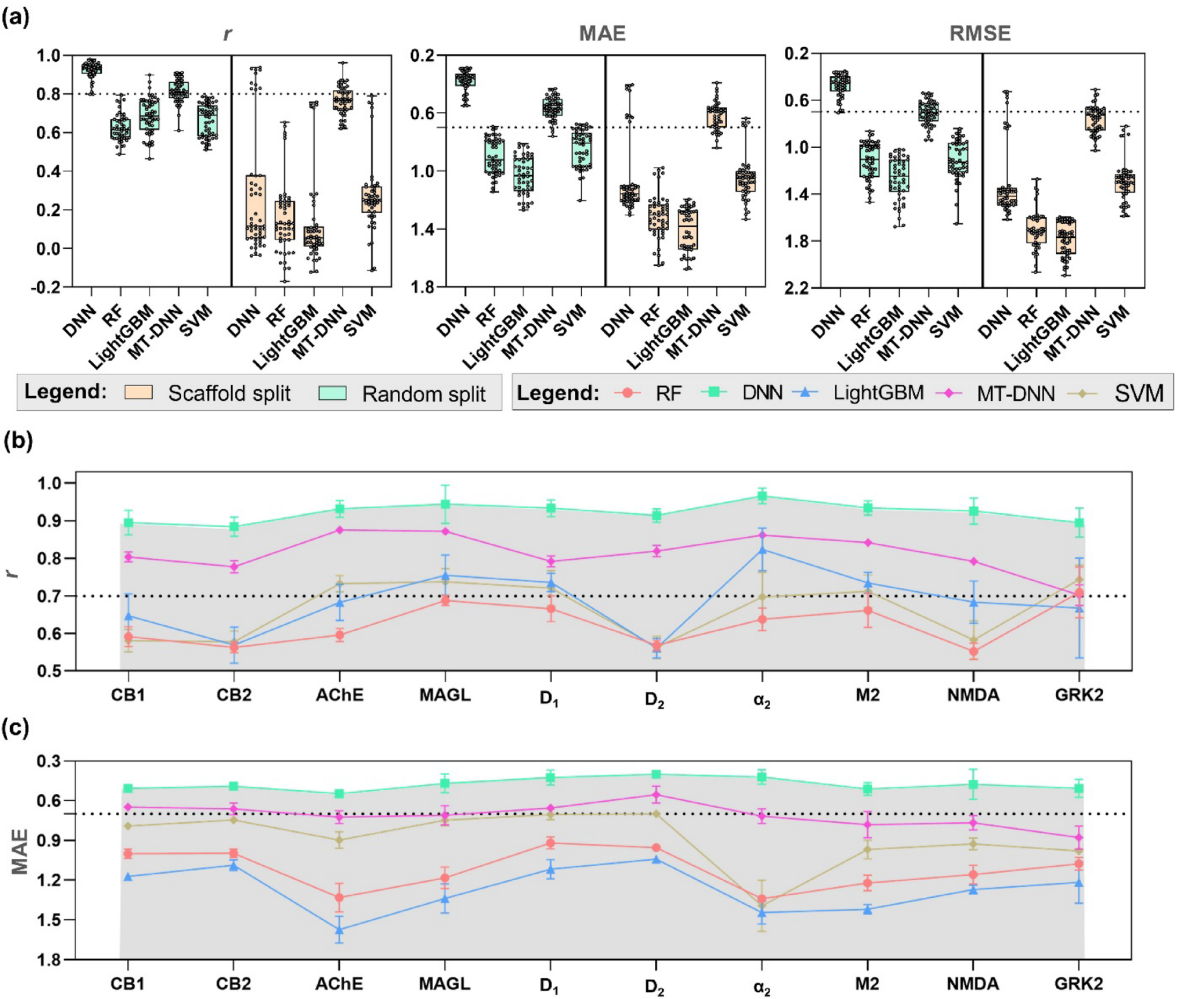
**a** Boxplots showcasing various machine learning methods’ global test set performance using scaffold and random data splits. The error bar represents the SD of the average performance over multiple assays. Mean Pearson correlation coefficients **b** and root-mean-square errors **c** indicate predictive performance of machine learning models across different assays. The error bar represents the SD of the average performance over five splitting runs

Analyzing Fig. 4a, it is possible to conclude that, in the standard random split approach, the single-task DNN (ST-DNN) model demonstrated superior performance compared to the multitasking model. This result starkly contrasts the scaffold split approach (see Fig. 4a), which offers a more challenging and realistic evaluation of the model’s predictive power. Scaffold split ensures no Murcko scaffold overlap between the train and test sets. The MT-DNN model exhibited the least performance loss using the scaffold split approach, demonstrating its robustness and consistent performance across different scenarios. Despite the superiority of the ST-DNN model in the Random Split approach, the MT-DNN models for MIEs also demonstrated high predictive power across all tasks, with correlation coefficients ranging from 0.702 ± 0.05 to 0.88 ± 0.02, as illustrated in Fig. 4b. The MAE, shown in Fig. 4c, ranged from 0.88 ± 0.06 for the GRK2 task (G-protein-coupled receptor kinase 2) to 0.55 ± 0.02 for the D2 task (Dopamine D2 receptor). Notably, the superiority of the ST-DNN model in the standard Random Split approach is an exception. In most other scenarios, the multitask approach excels, highlighting its general suitability for handling heterogeneous datasets. Moreover, multitask models exhibit better predictive capacity in tasks with sparse or noisy data, such as MAGL (monoglyceride lipase) and GRK2. They achieve this by generalizing representations across related tasks and applying them to new and unseen data, making them less susceptible to overfitting than single-task models. The statistical performance of the models also suggests that high-quality information is included in the ChEMBL database and that it is suitable as a data source for modeling the MIEs and KEs studied in this work.

Additionally, the comparative learning curves presented in Fig. S4 of the Supplementary Material reinforce the role of hard parameter-sharing as an effective regularization strategy. The single-task FNN exhibits a more pronounced divergence between training and validation curves for both RMSE and loss, particularly during the early and intermediate training stages, signaling a higher tendency toward task-specific overfitting. In contrast, the multitask FNN displays smoother convergence patterns and reduced discrepancies between training and validation metrics, consistent with the stabilizing and variance-reducing effects of shared representations.

### Applicability domain

The initial dataset exhibited high sparsity (~ 89%), an intrinsic characteristic of biological screening experiments. To enable computational algorithm application, the matrix was converted to a dense format. Compounds underwent structural standardization through the selection of the largest molecular fragment using the RDKit package. Chemical representation was encoded using Extended Connectivity Fingerprints (ECFP6; radius = 2, 1024 bits), followed by structural dissimilarity calculation based on the Tanimoto coefficient, resulting in a precomputed distance matrix for similarity analysis and Applicability Domain (AD) definition.

Chemical space projection into reduced dimensions was performed using the t-distributed Stochastic Neighbor Embedding (t-SNE) [39] algorithm. A systematic parametric analysis encompassed perplexity values of 10, 20, 30, 40, and 50, covering the established range for medium-sized molecular sets (up to 10⁴ compounds), with three random initialization replicates (seeds 0, 42, and 99) for each configuration [40–42]. The remarkable similarity observed among projections, regardless of the parameters used, corroborates the high density and local homogeneity inherent to the investigated chemical space (Fig. S5a, Supplementary Material). Quantitative validation employed multiple metrics: (i) trustworthiness, quantifying local neighborhood preservation through comparison between original and projected spaces [43]; (ii) Procrustes disparity, assessing geometric stability between projections through optimal transformation superposition [44]; and (iii) silhouette score, measuring clustering structure coherence [45]. Complementary binary criteria (trustworthy_flag and stable_flag) were derived for automated identification of optimized configurations [46]. The complete results of this systematic optimization are summarized in Table 2.

**Table 2:** Quantitative evaluation of t-SNE projections across multiple parameter configurations

Perplexity	Seed	Trustworthiness	Procrustes_disparity	Silhouette	Trustworthy_flag	Stable_flag
10	99	0.99392	0.228475	0.325657	True	False
10	0	0.99387	–	0.325983	True	False
10	42	0.993831	0.175083	0.327525	True	False
20	42	0.994218	0.067182	0.344109	True	False
20	99	0.994078	0.088669	0.340385	True	False
20	0	0.994047		0.32273	True	False
30	99	0.993034	0.027608	0.337602	True	False
30	42	0.993029	0.027217	0.339105	True	False
30	0	0.992885		0.343086	True	False
40	42	0.992835	0.061698	0.336906	True	False
40	0	0.99251		0.337138	True	False
40	99	0.992464	0.046891	0.328462	True	False
50	0	0.992767		0.330297	True	False
50	42	0.992715	0.017237	0.336119	True	False
50	99	0.992216	0.020079	0.334948	True	False

Each combination of perplexity and random initialization (seed) was assessed using three complementary metrics: *trustworthiness* (local neighborhood preservation), *Procrustes disparity* (geometric stability across initializations), and *silhouette score* (cluster separation). Binary flags (*trustworthy_flag*, *stable_flag*) denote compliance with predefined reliability thresholds. Missing *Procrustes disparity* values correspond to reference projections used for alignment within each perplexity group.

Systematic analysis of t-SNE projections (Table 2) revealed high preservation of local neighborhoods, with trustworthiness values exceeding 0.992 across all executions, indicating that the algorithm consistently captures the local structure of the high-dimensional space, independent of initialization conditions. Separation between clusters, evaluated by the silhouette index, showed moderate values (0.322–0.344), suggesting that intermediate perplexities (20–30) promote slightly superior cluster distinction. In contrast, structural consistency between executions, measured by Procrustes disparity, demonstrated significant dependence on perplexity. Intermediate configurations resulted in low disparity (~ 0.027), whereas lower values exhibited greater instability (> 0.17), evidencing the trade-off between local preservation and projection reproducibility. The configuration with Perplexity = 20 and Seed = 42 was selected as representing the optimal compromise between local neighborhood preservation, cluster distinction, and structural consistency. Although all combinations met the local reliability criterion (trustworthy_flag = True), none achieved complete stability (stable_flag = False), reflecting the intrinsically stochastic nature of t-SNE. These findings indicate that while local representation of the chemical space is robust, interpretation of global patterns requires caution and should be supported by multiple initializations and additional quantitative metrics.

For Applicability Domain quantification, the k-Nearest Neighbors (k-NN) [47] algorithm was implemented on the Tanimoto distance matrix, where parameter k represents the number of nearest neighbors considered for each molecule. Determination of the optimal neighborhood parameter through exhaustive parametric sweep (k = 3, 5, …, 29) revealed that neighborhood scope expansion promotes a monotonic increase in mean distances, reflecting progressive inclusion of more dissimilar points (Fig. S6, Supplementary Material). Notably, the corresponding variance remained essentially constant across different k values, indicating homogeneity in the local density of the chemical space. This cohesive topology, characterized by the absence of extremely sparse or dense regions, justified the selection of a reduced value of k = 3 for AD definition, ensuring the domain is based on the structurally most relevant neighborhood.

**Fig. 5:**
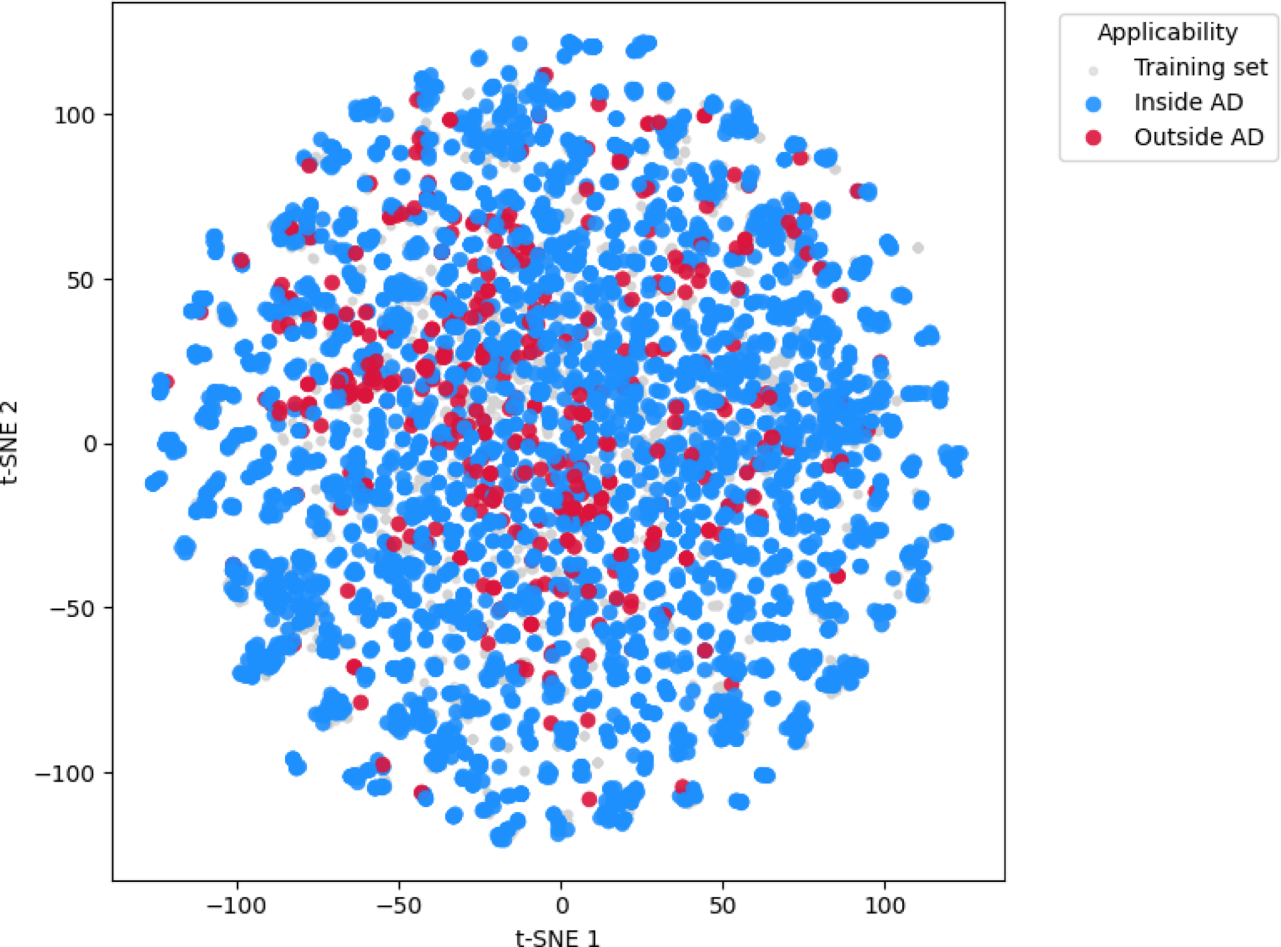
*t*-SNE projection of the chemical space based on the Tanimoto distance matrix. Grey points represent training compounds, blue points indicate test compounds Inside the AD, and red points correspond to those Outside AD

The operational threshold for the Applicability Domain was established at 0.3217, which demarcates the upper limit of the dense core of the distance distribution in the training space and comprises approximately 95% of the spatial distribution of the set (Fig. S5b, Supplementary Material). Molecules with distances exceeding this threshold are considered outside the applicability domain, and their predictions should be interpreted with caution. To further assess the spatial consistency of the applicability boundary, the chemical space was projected using t-SNE based on the precomputed Tanimoto distance matrix (Fig. 5). Training samples are shown in grey, while test samples are colored according to their Applicability Domain classification (blue = Inside AD; red = Outside AD).

In this representation, the training compounds form a continuous and compact manifold occupying the central region of the projection, whereas test molecules identified as Outside AD are dispersed toward the periphery. This spatial gradient, extending from a dense core of structurally interrelated compounds to outer regions of increasing dissimilarity, visually recapitulates the statistical cutoff derived from the k-NN distance distribution.Collectively, this spatial analysis substantiates the robustness of the adopted AD definition: the t-SNE topology visually corroborates the continuity and compactness of the reliable prediction region, while the peripheral dispersion of atypical structures highlights the inherent limitations of model generalization beyond the trained chemical manifold.

### Model explainability

Due to their complex architectural structures, robust methods such as neural networks function as “black boxes” they lack a native way to explain how results are achieved. This lack of transparency arises from the many layers and parameters involved and the nonlinear transformations applied to data during training. To reduce the opacity of the MT-DNN model, Shapley Additive Explanations (SHAP) were employed, which assess the influence of parameters based on cooperative game theory, enabling an equitable distribution of each parameter’s importance according to its contribution to the obtained response. SHAP can interpret activity predictions from complex DNN models [48].

Although the ST-DNN model demonstrated superior performance, the MT-DNN model was chosen for model explainability since it utilizes shared representations across multiple tasks, enhancing its ability to detect common patterns. The multitask architecture also reduces the likelihood of selecting irrelevant features, as the analysis is constrained to the chemical space of each task. In this sense, SHAP analysis facilitates the identification of features that maintain consistent importance across tasks, offering insights into the underlying relationships that the model has learned. This analysis elucidates how specific features contribute to various outcomes and highlights task-specific nuances. Consequently, comparing feature importance across tasks using SHAP informs targeted model design and feature engineering improvements.

### Global model diagnostics

**Fig. 6:**
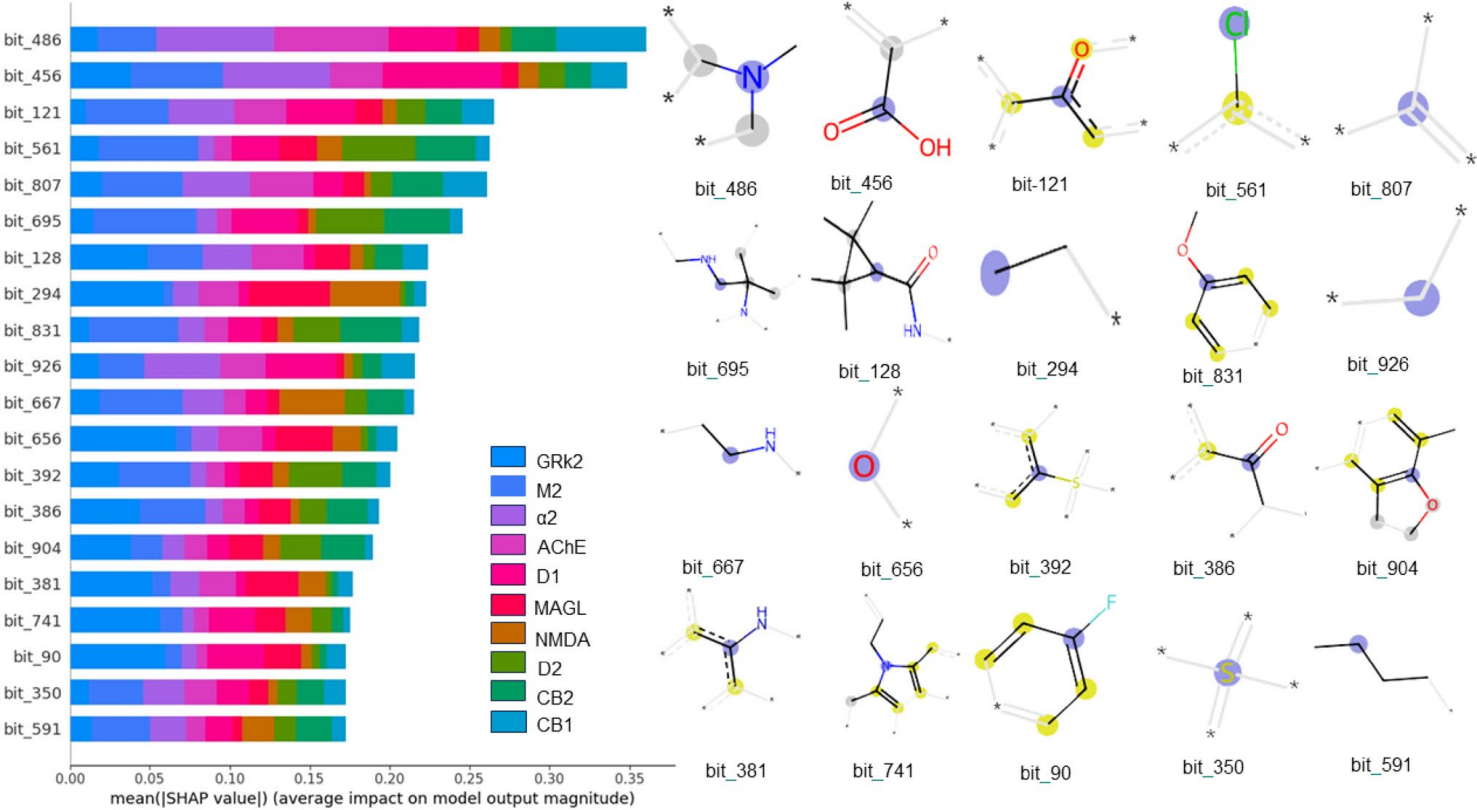
SHAP summary plot for the top 20 molecular fragments impacting the MT-DNN model’s predictions

SHAP values were used to analyze the MT-DNN model’s predictions, revealing the molecular fragments with the most significant influence on specific class assignments. The SHAP analysis in Fig. 6 shows a diverse distribution of the 20 essential fragments (bits) impacting the prediction towards MIEs and KEs. Tertiary amine, represented by bit 486, is the most crucial feature, impacting predominantly AChE, α2, and CB1, as indicated by its larger mean SHAP values in these tasks. In contrast, the carboxylic acid group, represented by bit 456, appears as the second most important feature, impacting predictions for the α2, D1, and M2 classes while having a lower impact on AChE predictions. As shown in Fig. 4, the effect of individual bits may vary significantly across different tasks, highlighting the unique contribution of each feature depending on the specific context. These results suggest that bit 486 is particularly relevant in compounds driving MIEs, whereas bit 456 plays a more prominent role in predicting KEs.

**Fig. 7:**
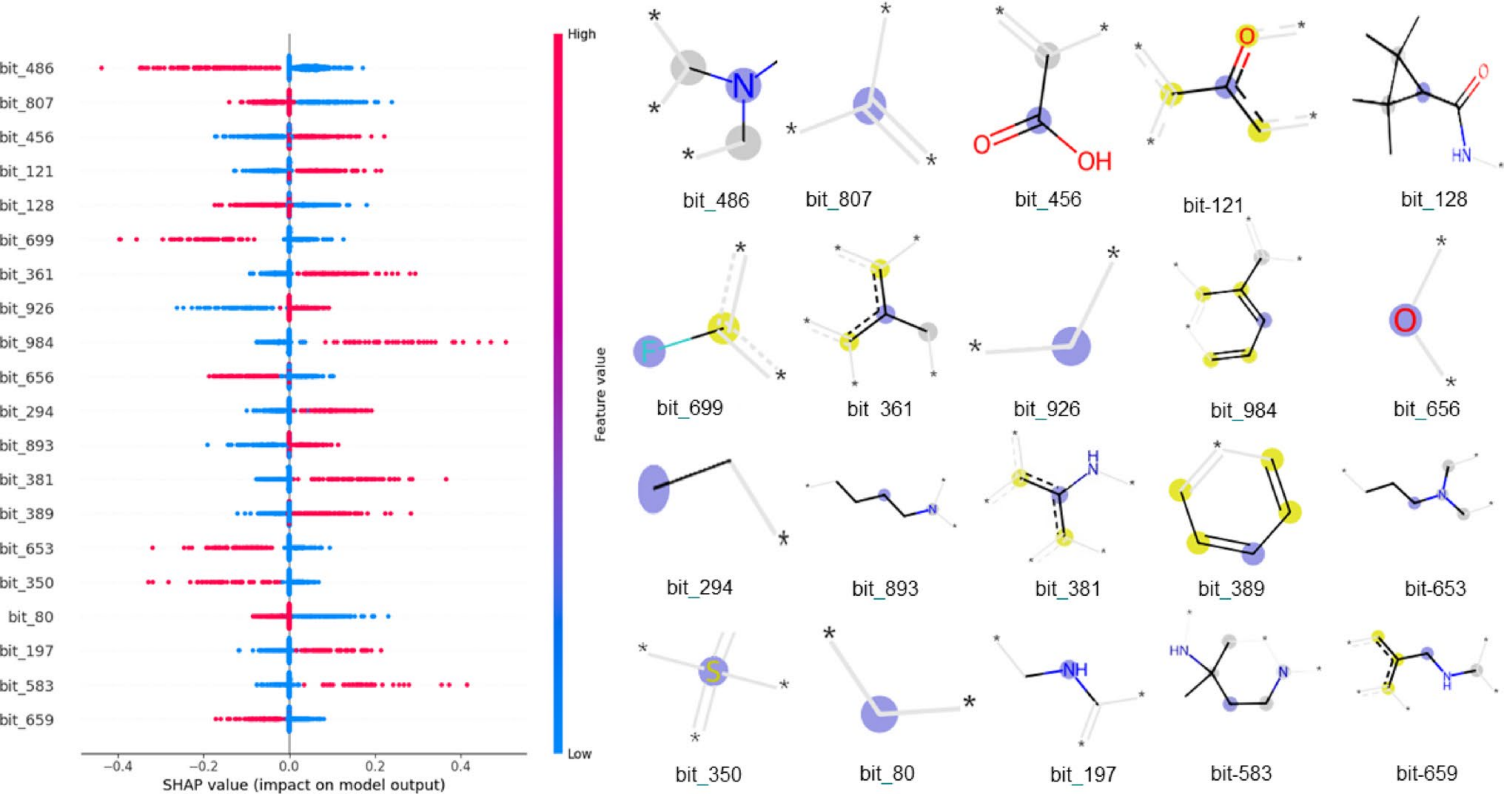
SHAP summary plot for the top 20 molecular fragments contributing the MT-DNN model’s predictions.

Subsequently, SHAP values were used to analyze the MT-DNN model’s predictions, providing insights into the molecular fragments that significantly influence the model’s output. Figure 7, presents a detailed insight into how the presence or absence of top 20 molecular fragments impacts the model’s predictions across multiple tasks, helping to elucidate the underlying molecular features that drive activity within the multitask learning framework. SHAP values along the x-axis represent the contribution of each feature to the predicted activity, with positive values indicating an increase and negative values indicating a decrease in activity. The plot reveals that the presence of specific fragments (red points) tends to enhance or reduce the predicted activity, while their absence (blue points) often has a neutral or opposite effect. For instance, the fragment represented by bits 486 (tertiary amine) consistently contributes negatively to the model’s predictions when present. The absence of this fragment has little positive contribution, as shown by the blue points centered near zero, suggesting that its presence is a key driver of higher predicted activity. Similarly, bits 807 and 128 (alkyl and tetramethylcyclopropane-1-carboxamide, respectively) contributes negatively to the prediction when present, while its absence leads to similar positive effect. In contrast, other fragments represented by bits 456, 121, 361, and 984 exhibit positive contributions when present. The red points on the plot’s right side indicate that their absence increases predicted activity. In contrast, its absence, represented by blue points, has a neutral or slightly negative impact on predictions.

### Task-based model diagnostics

The contributions of the ECFP4 features were mapped onto compounds to assess whether the MT-DNN model’s activity predictions were grounded in relevant molecular interactions. SHAP was used to illustrate the specific regions within each compound most likely responsible for biological activity related to MIEs and KEs. The local SHAP value calculations quantify the feature contributions that explain the predicted pIC_50_ values. By combining the base value of the model with the individual feature contributions, we could predict activity in terms of pIC_50_ for specific targets. This approach enables the identification and visualization of structural patterns within the compounds that influence these predictions, helping to highlight features that may be critical in determining activity across various MIE and KE targets.

**Fig. 8:**
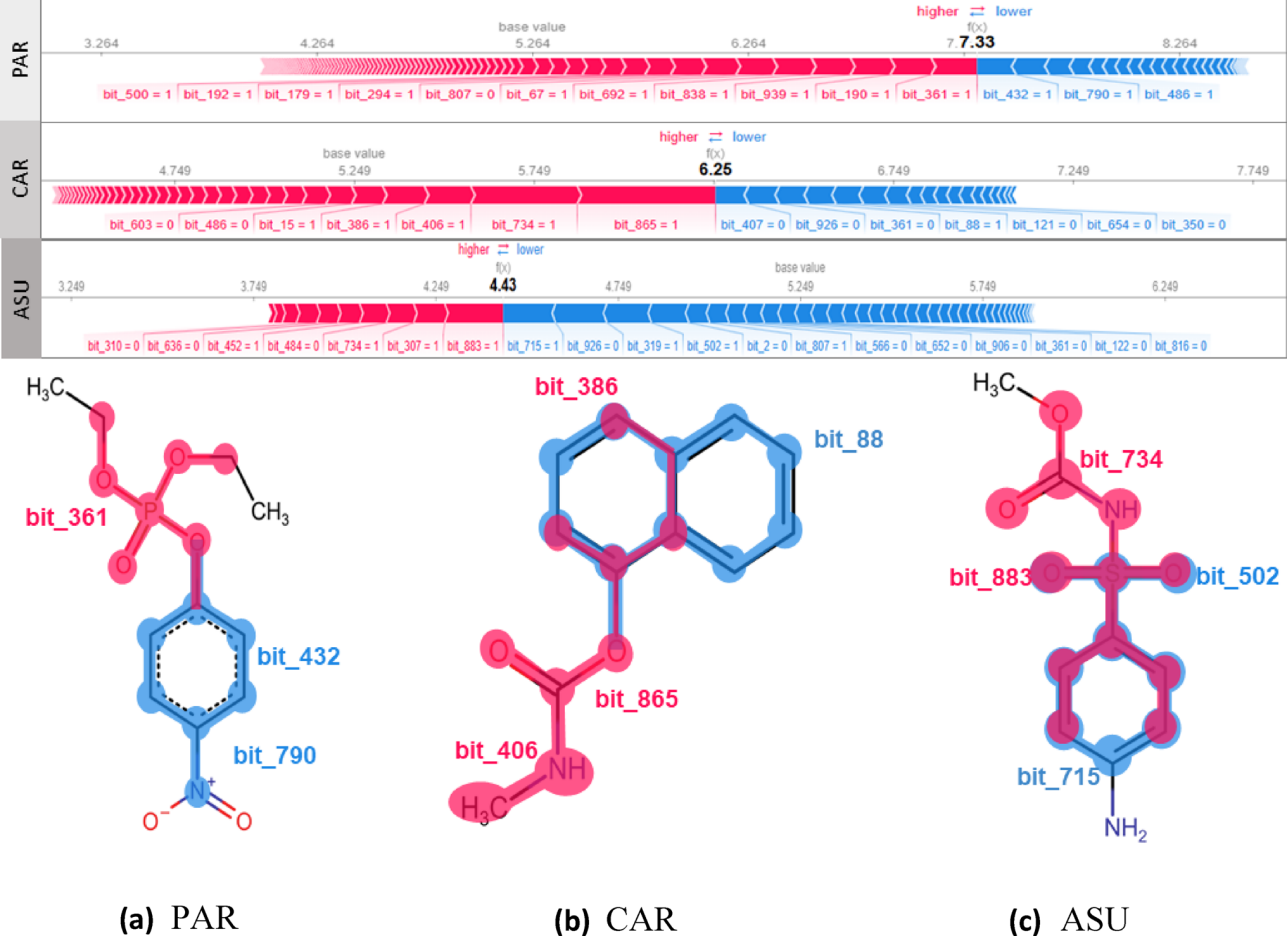
SHAP values for ECFP4 features. Displayed neurotoxicants: paraoxon **a** Carbaryl **b** and Asulam **c** represented using ECFP4, highlighting regions that contribute positively and negatively to the interaction with acetylcholinesterase

Figure 8 presents the contributions of the ECFP4 descriptor’s structural features to the predicted activity of three compounds widely used as insecticides or herbicides in agriculture — paraoxon (PAR, CAS: 311-45-5), carbaryl (CAR, CAS: 63-25-2), and asulam (ASUL, CAS: 3337-71-1). Although all play relevant roles in pest control, only PAR (Fig. 8a) and CAR (Fig. 8b) are recognized as active against the enzyme acetylcholinesterase (AChE), whereas ASUL (Fig. 8c)shows no significant affinity for this molecular target. The predictions were generated using the MT-DNN model and interpreted via SHAP analysis, with a particular focus on AChE, one of the selected biological targets, owing to its critical neurophysiological role—since its inhibition disrupts cholinergic homeostasis and impairs essential neural functions [49, 50].

The choice of the multitask model is justified by the heterogeneous nature of the dataset, which comprises ten biological targets with varying numbers of annotated compounds. In this context, the multitask learning approach enables information sharing across related tasks, supporting the development of more generalizable molecular representations and reducing the risk of overfitting in tasks with limited data. Although the single-task model showed better predictive performance for AChE specifically, the MT-DNN was adopted for SHAP analysis due to its higher stability and its ability to capture relevant structural patterns in an integrated manner across tasks, which is particularly advantageous for interpretability purposes [51].

As illustrated in Fig. 8a, the phosphate group (bit_361) present in the structure of PAR shows a strong positive contribution to the predicted inhibitory activity against AChE, consistent with the classical mechanism of action described in the literature, in which strongly electrophilic groups form covalent bonds with nucleophilic residues in the enzyme’s catalytic site, notably the active serine [52]. Similarly, the CAR (Fig. 8b) and ASUL (Fig. 8c) compounds contain carbamate-type substructures, represented by bits bit_865 and bit_734, respectively, commonly associated with carbamylation of the AChE active site through a nucleophilic attack on the carbonyl carbon, leading to stable enzyme inhibition [53].

However, the detailed analysis of individual structural contributions reveals a critical distinction in the case of ASUL. Although some bits associated with the carbamate substructure exhibit local positive contributions to the activity prediction — highlighted by the pink regions in Fig. 8c — these fragments are not, in combination, sufficient to raise the predicted pIC_50_ value to a threshold compatible with relevant pharmacological activity against AChE. The MT-DNN model predicts a pIC_50_ value of 4.43 for ASUL, classifying it as inactive under the adopted criteria. This prediction is in full agreement with experimental toxicological data available in the literature, which do not report neurotoxic effects associated with ASUL [54]. Thus, although the model identifies local contributions from activating bits linked to the carbamate moiety, the overall fingerprint vector generated from the ASUL structure does not encode sufficient information to predict effective interaction with AChE. The consistency between predictive results reinforces the accuracy and robustness of the interpretable model employed in this analysis.

**Fig. 9:**
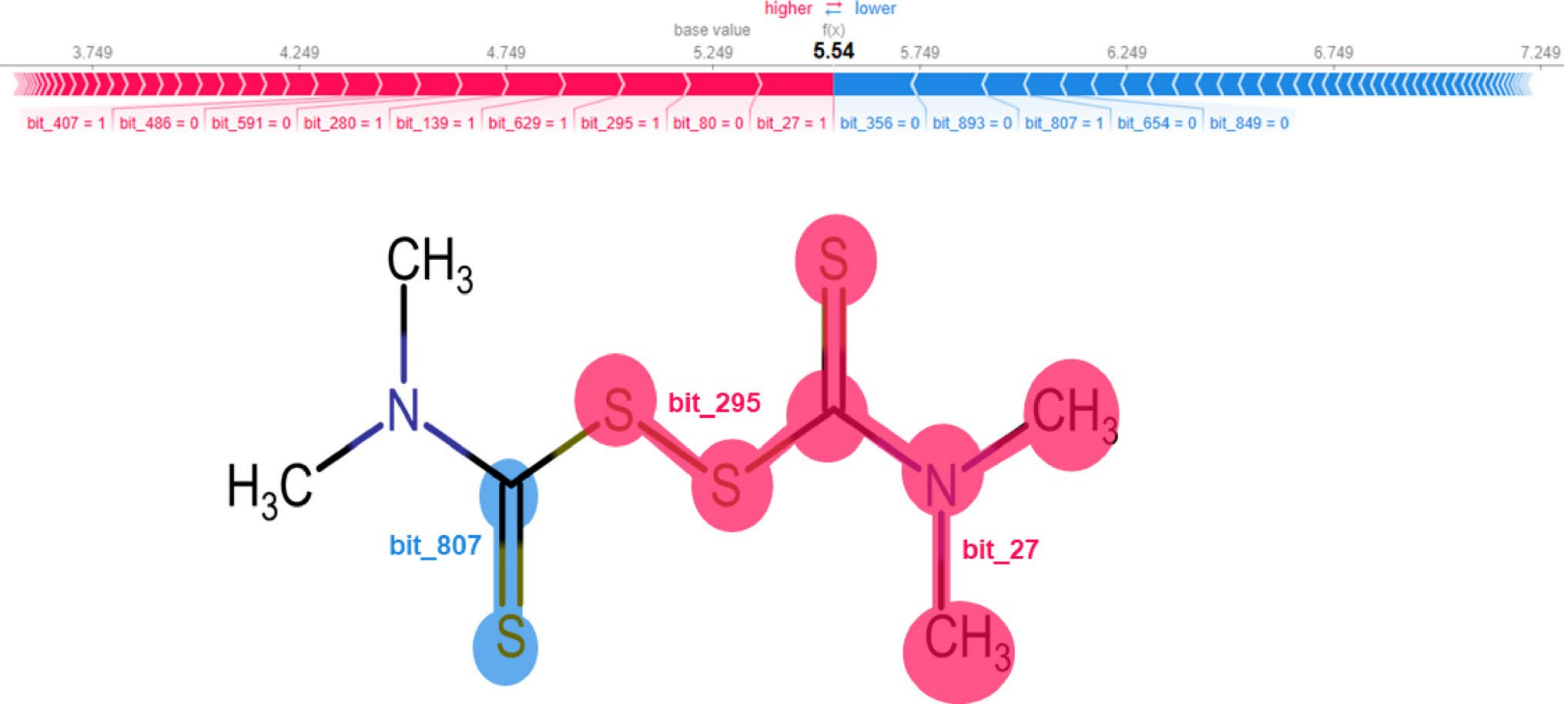
The chemical structure of disulfiram was visually analyzed to identify specific regions potentially contributing to its observed activity. In the visual representation, regions with positive contributions are highlighted in pink, indicating that these features positively influence disulfiram’s interaction with the MAGL task

Monoacylglycerol Lipase (MAGL) was additionally selected as a representative target to evaluate the model’s capacity to generalize across tasks with distinct data regimes, given its markedly smaller dataset (661 compounds) compared to that of acetylcholinesterase (AChE), which comprises 4,693 entries. To examine the performance and interpretability of the MT-DNN model under such data-sparse conditions, SHAP analysis was employed to investigate the model’s prediction on disulfiram, a pharmacological agent commonly used in the management of alcohol dependence [55]. Disulfiram is known to inhibit MAGL, an enzyme integral to the regulation of the endocannabinoid system via hydrolysis of 2-arachidonoylglycerol [56]. Notably, the SHAP analysis (Fig. 9) revealed a local positive contribution from the disulfide bonding region (bit_295) to the predicted inhibitory activity. This finding is consistent with mechanistic studies suggesting that disulfiram’s inhibitory effect involves intramolecular disulfide interactions within the catalytic environment of MAGL [56]. Such alignment between the model’s attributions and established biochemical mechanisms underscores its ability to capture chemically relevant features, thereby reinforcing the reliability and mechanistic interpretability of the MT-DNN framework even in the context of limited data availability.

## Conclusion

In conclusion, our in silico framework enhances predictive accuracy and provides valuable mechanistic insights into the MIEs and KEs relevant to DNT. A key outcome was the construction of a structurally diverse dataset comprising 21,787 compounds from the ChEMBL database, encompassing a diverse array of bioassays related to neurotoxicity. Using this dataset, we developed models in both multi-task and single-task learning paradigms, with robust performance — achieving *r* values higher than 0.8 and RMSE lower than 0.6 across multiple tasks. Interpretable analysis, in turn, was conducted using the MT-DNN, given its ability to integrate shared information across tasks with different data volumes. The chemical space of the dataset, characterized by a predominant lipophilicity (LogP) near zero and a broad molecular weight distribution, encompasses a phyisicochemically diverse compound set. This diversity is crucial for the development of models with a wide applicability domain and robust generalization potential to structurally heterogeneous molecules. Additionally, the applicability domain was evaluated using t-SNE and k-NN for outlier detection, identifying regions of high applicability where predictions are more reliable, and areas of lower applicability, indicating caution for compounds outside the training set’s boundaries.

While the study did not directly predict DNT outcomes, the individual predictions of MIEs and KEs are critical components that can be incorporated into broader IATA. These predictions offer important evidence for understanding the initial molecular interactions that may lead to neurotoxicity, supporting more informed regulatory and scientific decision-making. Moreover, this methodology lays the groundwork for the design of safer chemical alternatives by identifying potential early-stage interactions of concern. As higher-quality data becomes available and additional MIEs are modeled, the predictive performance of these QSAR models is expected to improve, further strengthening their applicability in comprehensive chemical toxicity assessments.

## Supplementary Information

Below is the link to the electronic supplementary material.

Supplementary Material 1

## Data Availability

No datasets were generated or analysed during the current study.
