# Supplementary material for: Deep learning-enhanced QSAR modeling for predicting developmental neurotoxicity based on molecular initiating events from adverse outcome pathways
Source: Mol Divers. 2026 Jan 23;30(2):2627–42. doi: 10.1007/s11030-025-11454-6 (PMC13139284; doi:10.1007/s11030-025-11454-6)
Supplement: Supplementary file 1 — Supplementary Material 1 [file 11030_2025_11454_MOESM1_ESM.docx]

**Supporting Information for Original article**

**Deep Learning-Enhanced QSAR Modeling for Predicting Developmental Neurotoxicity Based on Molecular Initiating Events from Adverse Outcome Pathways**

Eufrásia de Sousa Pereira ^1*^, Vinícius Alexandre Fiaia Costa,^1^ Eder Soares de Almeida Santos,^1^ Bruno Junior Neves ^1,^*

^1^ Laboratory of Cheminformatics, Faculty of Pharmacy, Federal University of Goiás, Goiânia, Brazil

*Author for correspondence: E-mail addresses: [brunoneves@ufg.br](mailto:brunoneves@ufg.br) (Bruno Junior Neves), [efrsousa@gmail.com](mailto:efrsousa@gmail.com) (Eufrásia de Sousa Pereira)

Journal: Molecular Diversity


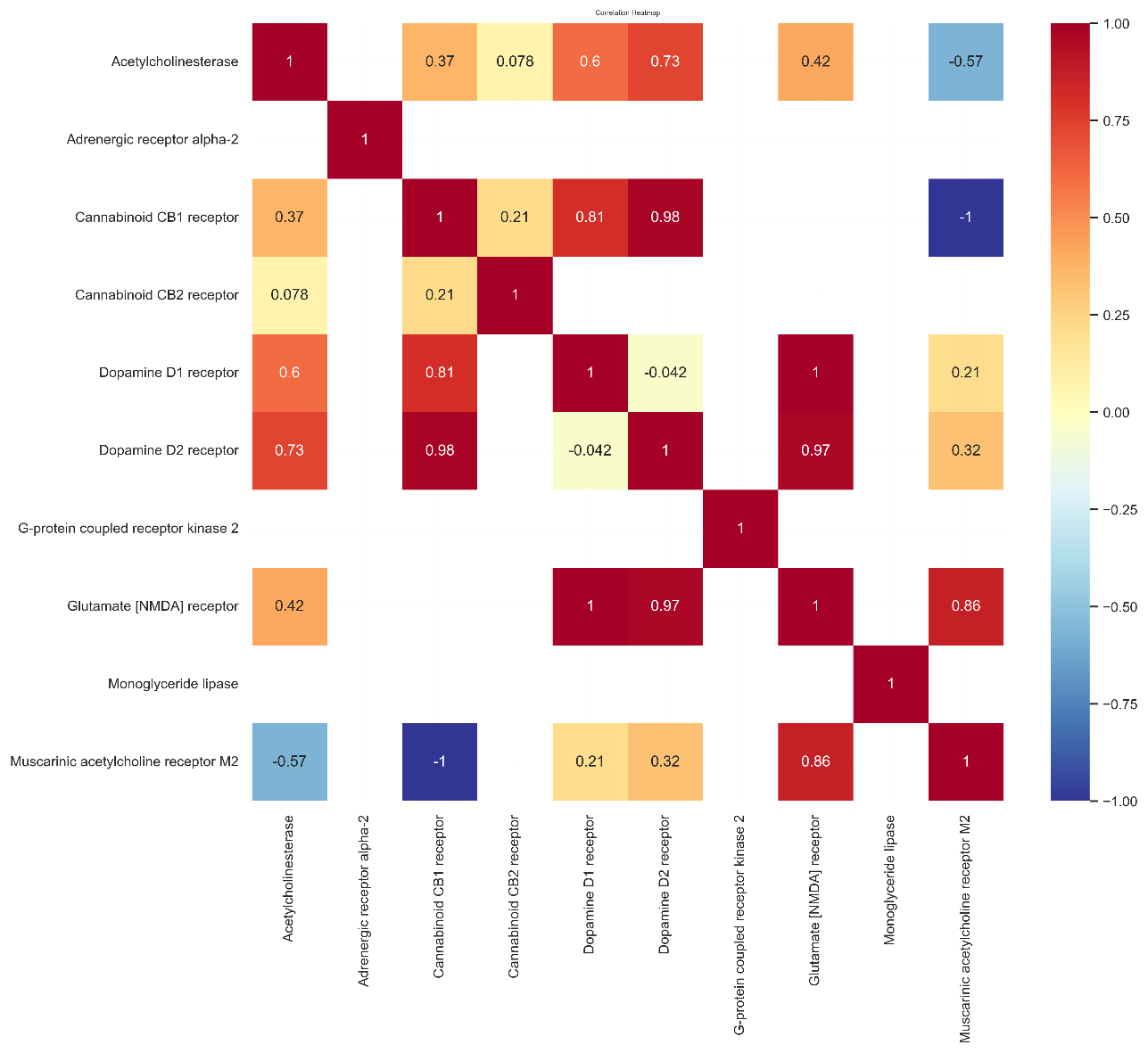


**Fig. S1.** Heat maps showing the correlation among the pIC_50_ values of the ten tasks.


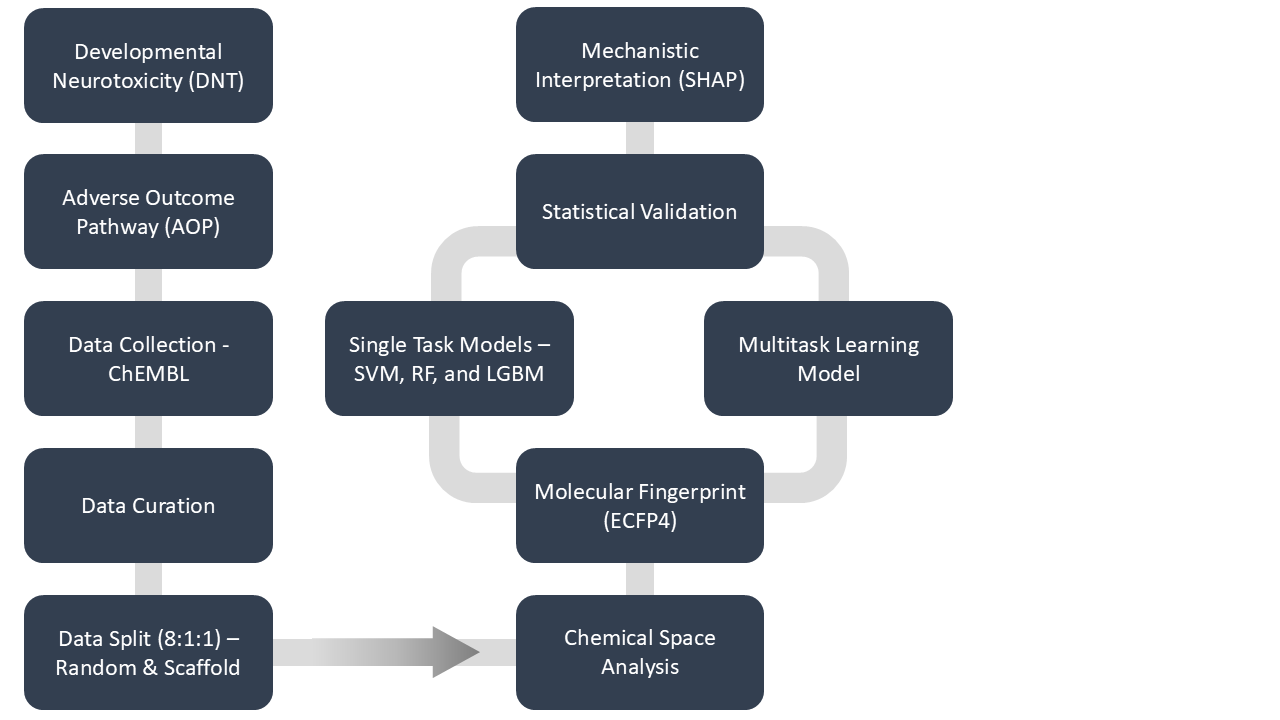


**Fig. S2.** Schematic workflow of the Deep Learning-Enhanced QSAR modeling framework for predicting developmental neurotoxicity, integrating AOP concepts, data curation, model development, and interpretability analysis.

**Table S1**. Summarized statistical characteristics of multitask and single-task models developed using random split.

| **Assay** | **FNN** | | | **RF** | | | **LightGBM** | | | **MT-FNN** | | | **SVM** | | |
| --- | --- | --- | --- | --- | --- | --- | --- | --- | --- | --- | --- | --- | --- | --- | --- |
|  | ***r*** | **MAE** | **RMSE** | ***r*** | **MAE** | **RMSE** | ***r*** | **MAE** | **RMSE** | ***r*** | **MAE** | **RMSE** | ***r*** | **MAE** | **RMSE** |
| CB1 | 0.881 ± 0.008 | 0.415 ± 0.015 | 0.529  ±  0.035 | 0.711 ± 0.009 | 0.958 ± 0.051 | 1.239  ±  0.198 | 0.691 ± 0.308 | 1.06  ± 0.036 | 1.317  ±  0.118 | 0.804 ± 0.018 | 0.524 ± 0.034 | 0.648  ± 0.042 | 0.581 ± 0.03 | 0.79  ± 0.013 | 1.016  ± 0.007 |
| CB2 | 0.869 ± 0.082 | 0.605 ± 0.031 | 0.736 ± 0.126 | 0.802 ± 0.38 | 1.096 ± 0.015 | 1.579 ± 0.345 | 0.96 ± 0.376 | 1.168 ± 0.057 | 1.735 ± 0.515 | 0.778 ± 0.022 | 0.534 ± 0.015 | 0.662 ± 0.027 | 0.578 ± 0.029 | 0.744 ± 0.019 | 0.95 ± 0.024 |
| MAGL | 0.836 ± 0.053 | 0.693 ± 0.079 | 0.825 ± 0.077 | 0.789 ± 0.234 | 0.88 ± 0.051 | 1.164 ± 0.174 | 0.804 ± 0.286 | 1.164 ± 0.076 | 1.472 ± 0.17 | 0.872 ± 0.046 | 0.564 ± 0.08 | 0.71 ± 0.096 | 0.733 ± 0.022 | 0.897 ± 0.061 | 1.197 ± 0.073 |
| AchE | 0.829 ± 0.035 | 0.75 ± 0.052 | 0.898 ± 0.097 | 0.777 ± 0.193 | 0.879 ± 0.039 | 1.09 ± 0.094 | 0.706 ± 0.323 | 1.062 ± 0.169 | 1.306 ± 0.197 | 0.876 ± 0.02 | 0.566 ± 0.035 | 0.724 ± 0.053 | 0.738 ± 0.035 | 0.747 ± 0.041 | 1.14 ± 0.071 |
| D_1_ | 0.813 ± 0.026 | 0.603 ± 0.054 | 0.727 ± 0.093 | 0.762 ± 0.138 | 0.782 ± 0.024 | 0.967 ± 0.043 | 0.752 ± 0.278 | 0.944 ± 0.046 | 1.214 ± 0.199 | 0.792 ± 0.033 | 0.53 ± 0.038 | 0.654 ± 0.052 | 0.721 ± 0.047 | 0.704 ± 0.037 | 0.891 ± 0.048 |
| D_2_ | 0.788 ± 0.074 | 0.526 ± 0.016 | 0.595 ± 0.092 | 0.646 ± 0.22 | 0.805 ± 0.01 | 1.007 ± 0.046 | 0.591 ± 0.269 | 0.952 ± 0.025 | 1.134 ± 0.028 | 0.82 ± 0.015 | 0.448 ± 0.016 | 0.554 ± 0.016 | 0.563 ± 0.03 | 0.698 ± 0.022 | 0.936 ± 0.028 |
| α_2_ | 0.799 ± 0.054 | 0.556 ± 0.007 | 0.632 ± 0.075 | 0.676 ± 0.187 | 0.827 ± 0.036 | 1.023 ± 0.032 | 0.651 ± 0.311 | 1.011 ± 0.018 | 1.239 ± 0.113 | 0.862 ± 0.046 | 0.556 ± 0.061 | 0.716 ± 0.07 | 0.697 ± 0.066 | 1.393 ± 0.191 | 0.978 ± 0.162 |
| M_2_ | 0.747 ± 0.072 | 0.47 ± 0.011 | 0.545 ± 0.092 | 0.646 ± 0.17 | 0.74 ± 0.019 | 0.931 ± 0.028 | 0.546 ± 0.294 | 0.858 ± 0.018 | 1.089 ± 0.034 | 0.842 ± 0.041 | 0.634 ± 0.068 | 0.78 ± 0.086 | 0.712 ± 0.043 | 0.967 ± 0.068 | 1.205 ± 0.072 |
| NMDA | 0.844 ± 0.076 | 0.787 ± 0.047 | 0.96 ± 0.045 | 0.756 ± 0.191 | 0.975 ± 0.035 | 1.194 ± 0.03 | 0.788 ± 0.384 | 1.266 ± 0.043 | 1.649 ± 0.295 | 0.792 ± 0.021 | 0.616 ± 0.036 | 0.766 ± 0.045 | 0.583 ± 0.051 | 0.925 ± 0.043 | 1.161 ± 0.037 |
| GRK2 | 0.838 ± 0.009 | 0.633 ± 0.02 | 0.787 ± 0.059 | 0.758 ± 0.362 | 1.02 ± 0.028 | 1.506 ± 0.284 | 0.73 ± 0.533 | 1.27 ± 0.054 | 1.89 ± 0.539 | 0.702 ± 0.055 | 0.67 ± 0.063 | 0.878 ± 0.061 | 0.744 ± 0.038 | 0.978 ± 0.016 | 1.194 ± 0.028 |

**Table S2**. Summarized statistical characteristics of multitask and single-task regression models developed using scaffold-split

| **Assay** | **DNN** | | | **RF** | | | **LightGBM** | | | **MT-DNN** | | | **SVM** | | |
| --- | --- | --- | --- | --- | --- | --- | --- | --- | --- | --- | --- | --- | --- | --- | --- |
|  | ***r*** | **MAE** | **RMSE** | ***r*** | **MAE** | **RMSE** | ***r*** | **MAE** | **RMSE** | ***r*** | **MAE** | **RMSE** | ***r*** | **MAE** | **RMSE** |
| CB1 | 0.099 ± 0.014 | 1.135 ± 0.03 | 1.402 ± 0.042 | 0.076 ± 0.031 | 1.374 ± 0.04 | 1.768 ± 0.066 | 0.091 ± 0.029 | 1.558 ± 0.042 | 1.925 ± 0.07 | 0.762 ± 0.03 | 0.584 ± 0.008 | 0.712 ± 0.014 | 0.215 ± 0.047 | 1.041 ± 0.039 | 1.267 ± 0.04 |
| CB2 | 0.012 ± 0.033 | 1.236 ± 0.044 | 1.515 ± 0.053 | -0.104 ± 0.039 | 1.595 ± 0.044 | 1.965 ± 0.07 | 0.02 ± 0.013 | 1.627 ± 0.041 | 1.995 ± 0.07 | 0.726 ± 0.027 | 0.56 ± 0.02 | 0.684 ± 0.025 | -0.006 ± 0.091 | 1.247 ± 0.073 | 1.494 ± 0.065 |
| MAGL | 0.297 ± 0.025 | 1.19 ± 0.015 | 1.478 ± 0.021 | 0.127 ± 0.034 | 1.255 ± 0.035 | 1.641 ± 0.056 | 0.047 ± 0.038 | 1.298 ± 0.041 | 1.675 ± 0.064 | 0.71 ± 0.096 | 0.656 ± 0.058 | 0.804 ± 0.053 | 0.271 ± 0.05 | 1.021 ± 0.043 | 1.244 ± 0.042 |
| AchE | 0.925 ± 0.011 | 0.421 ± 0.017 | 0.56 ± 0.039 | 0.608 ± 0.034 | 1.008 ± 0.025 | 1.364 ± 0.062 | 0.742 ± 0.011 | 1.269 ± 0.038 | 1.65 ± 0.061 | 0.83 ± 0.035 | 0.658 ± 0.066 | 0.856 ± 0.104 | 0.208 ± 0.031 | 1.206 ± 0.054 | 1.525 ± 0.043 |
| D_1_ | 0.009 ± 0.041 | 1.148 ± 0.046 | 1.4 ± 0.064 | 0.067 ± 0.024 | 1.356 ± 0.039 | 1.753 ± 0.065 | 0.004 ± 0.046 | 1.477 ± 0.042 | 1.851 ± 0.069 | 0.794 ± 0.051 | 0.582 ± 0.114 | 0.716 ± 0.135 | 0.314 ± 0.02 | 1.05 ± 0.039 | 1.254 ± 0.045 |
| D_2_ | 0.112 ± 0.023 | 1.177 ± 0.052 | 1.421 ± 0.063 | 0.235 ± 0.011 | 1.389 ± 0.044 | 1.777 ± 0.07 | 0.041 ± 0.042 | 1.472 ± 0.043 | 1.847 ± 0.07 | 0.748 ± 0.021 | 0.502 ± 0.039 | 0.622 ± 0.047 | 0.264 ± 0.033 | 1.037 ± 0.045 | 1.246 ± 0.046 |
| α_2_ | 0.354 ± 0.035 | 1.133 ± 0.025 | 1.402 ± 0.042 | 0.232 ± 0.023 | 1.23 ± 0.032 | 1.636 ± 0.057 | -0.075 ± 0.042 | 1.242 ± 0.035 | 1.682 ± 0.055 | 0.856 ± 0.064 | 0.57 ± 0.13 | 0.71 ± 0.143 | 0.29 ± 0.029 | 1.051 ± 0.052 | 1.33 ± 0.056 |
| M_2_ | 0.052 ± 0.015 | 1.246 ± 0.025 | 1.545 ± 0.044 | -0.022 ± 0.003 | 1.415 ± 0.045 | 1.805 ± 0.069 | 0.03 ± 0.05 | 1.56 ± 0.042 | 1.927 ± 0.07 | 0.792 ± 0.031 | 0.684 ± 0.059 | 0.828 ± 0.085 | 0.157 ± 0.046 | 1.128 ± 0.058 | 1.351 ± 0.057 |
| NMDA | 0.836 ± 0.014 | 0.629 ± 0.017 | 0.818 ± 0.02 | 0.126 ± 0.014 | 1.242 ± 0.035 | 1.656 ± 0.056 | 0.103 ± 0.031 | 1.253 ± 0.035 | 1.657 ± 0.056 | 0.694 ± 0.075 | 0.72 ± 0.094 | 0.884 ± 0.111 | 0.742 ± 0.034 | 0.666 ± 0.02 | 0.873 ± 0.035 |
| GRK2 | 0.117 ± 0.062 | 1.144 ± 0.042 | 1.425 ± 0.062 | 0.261 ± 0.023 | 1.256 ± 0.04 | 1.634 ± 0.063 | 0.26 ± 0.025 | 1.292 ± 0.041 | 1.668 ± 0.065 | 0.752 ± 0.088 | 0.664 ± 0.075 | 0.788 ± 0.09 | 0.272 ± 0.062 | 1.022 ± 0.052 | 1.293 ± 0.046 |

**Table S3**. Summarizes statistical characteristics of the validation set multitasking and single-task regression models developed using random split

| **Assay** | **DNN** | | | **RF** | | | **LightGBM** | | | **MT-DNN** | | | **SVM** | | |
| --- | --- | --- | --- | --- | --- | --- | --- | --- | --- | --- | --- | --- | --- | --- | --- |
|  | ***r*** | **MAE** | **RMSE** | ***r*** | **MAE** | **RMSE** | ***r*** | **MAE** | **RMSE** | ***r*** | **MAE** | **RMSE** | ***r*** | **MAE** | **RMSE** |
| CB1 | 0.888 ± 0.052 | 0.457 ± 0.063 | 0.57 ± 0.074 | 0.586 ± 0.061 | 0.973 ± 0.054 | 1.174 ± 0.069 | 0.551 ± 0.031 | 1.054 ± 0.034 | 1.259 ± 0.031 | 0.804 ± 0.027 | 0.55 ± 0.042 | 0.67 ± 0.051 | 0.599 ± 0.021 | 0.79 ± 0.013 | 1.016 ± 0.007 |
| CB2 | 0.889 ± 0.041 | 0.636 ± 0.064 | 0.831 ± 0.102 | 0.6 ± 0.03 | 1.101 ± 0.018 | 1.436 ± 0.028 | 0.708 ± 0.075 | 1.193 ± 0.074 | 1.531 ± 0.082 | 0.808 ± 0.023 | 0.5 ± 0.027 | 0.62 ± 0.037 | 0.562 ± 0.03 | 0.744 ± 0.019 | 0.95 ± 0.024 |
| MAGL | 0.832 ± 0.029 | 0.669 ± 0.075 | 0.812 ± 0.077 | 0.69 ± 0.049 | 0.899 ± 0.061 | 1.114 ± 0.079 | 0.664 ± 0.035 | 1.154 ± 0.069 | 1.39 ± 0.109 | 0.872 ± 0.021 | 0.572 ± 0.06 | 0.702 ± 0.069 | 0.716 ± 0.007 | 0.949 ± 0.01 | 1.22 ± 0.019 |
| AchE | 0.615 ± 0.13 | 0.877 ± 0.098 | 1.049 ± 0.09 | 0.68 ± 0.058 | 0.882 ± 0.035 | 1.064 ± 0.046 | 0.694 ± 0.137 | 1.055 ± 0.147 | 1.248 ± 0.156 | 0.868 ± 0.01 | 0.58 ± 0.018 | 0.76 ± 0.028 | 0.737 ± 0.021 | 0.747 ± 0.041 | 1.14 ± 0.071 |
| D_1_ | 0.786 ± 0.04 | 0.649 ± 0.023 | 0.804 ± 0.029 | 0.698 ± 0.02 | 0.781 ± 0.021 | 0.966 ± 0.042 | 0.641 ± 0.033 | 0.961 ± 0.053 | 1.152 ± 0.07 | 0.812 ± 0.038 | 0.508 ± 0.054 | 0.626 ± 0.053 | 0.694 ± 0.03 | 0.704 ± 0.037 | 0.891 ± 0.048 |
| D_2_ | 0.799 ± 0.032 | 0.516 ± 0.025 | 0.629 ± 0.029 | 0.556 ± 0.017 | 0.812 ± 0.017 | 0.998 ± 0.03 | 0.469 ± 0.048 | 0.94 ± 0.034 | 1.119 ± 0.038 | 0.822 ± 0.013 | 0.454 ± 0.021 | 0.566 ± 0.025 | 0.546 ± 0.02 | 0.698 ± 0.022 | 0.936 ± 0.028 |
| α_2_ | 0.828 ± 0.02 | 0.531 ± 0.026 | 0.635 ± 0.024 | 0.586 ± 0.019 | 0.823 ± 0.033 | 1.021 ± 0.033 | 0.467 ± 0.042 | 1.016 ± 0.019 | 1.191 ± 0.016 | 0.882 ± 0.038 | 0.52 ± 0.077 | 0.656 ± 0.111 | 0.685 ± 0.029 | 0.978 ± 0.162 | 1.393 ± 0.191 |
| M_2_ | 0.778 ± 0.023 | 0.476 ± 0.014 | 0.586 ± 0.013 | 0.566 ± 0.013 | 0.74 ± 0.016 | 0.94 ± 0.024 | 0.415 ± 0.037 | 0.86 ± 0.017 | 1.074 ± 0.013 | 0.816 ± 0.011 | 0.67 ± 0.022 | 0.814 ± 0.041 | 0.677 ± 0.034 | 0.967 ± 0.068 | 1.205 ± 0.072 |
| NMDA | 0.794 ± 0.023 | 0.769 ± 0.065 | 0.935 ± 0.072 | 0.672 ± 0.011 | 0.955 ± 0.053 | 1.175 ± 0.057 | 0.629 ± 0.049 | 1.258 ± 0.041 | 1.509 ± 0.034 | 0.806 ± 0.035 | 0.59 ± 0.065 | 0.734 ± 0.074 | 0.6 ± 0.035 | 0.925 ± 0.043 | 1.161 ± 0.037 |
| GRK2 | 0.815 ± 0.025 | 0.662 ± 0.03 | 0.845 ± 0.033 | 0.586 ± 0.02 | 1.021 ± 0.024 | 1.392 ± 0.107 | 0.545 ± 0.036 | 1.274 ± 0.048 | 1.659 ± 0.098 | 0.79 ± 0.051 | 0.618 ± 0.038 | 0.802 ± 0.056 | 0.537 ± 0.138 | 0.978 ± 0.016 | 1.194 ± 0.028 |

**Table S4**. Summarizes statistical characteristics of the train set multitasking and single-task regression models developed using random split

| **Assay** | **DNN** | | | **RF** | | | **LightGBM** | | | **MT-DNN** | | | **SVM** | | |
| --- | --- | --- | --- | --- | --- | --- | --- | --- | --- | --- | --- | --- | --- | --- | --- |
|  | ***r*** | **MAE** | **RMSE** | ***r*** | **MAE** | **RMSE** | ***r*** | **MAE** | **RMSE** | ***r*** | **MAE** | **RMSE** | ***r*** | **MAE** | **RMSE** |
| CB1 | 0.914 ± 0.014 | 0.416 ± 0.033 | 0.518 ± 0.046 | 0.588 ± 0.011 | 0.94 ± 0.016 | 1.145 ± 0.021 | 0.535 ± 0.018 | 1.086 ± 0.009 | 1.297 ± 0.009 | 0.946 ± 0.016 | 0.3 ± 0.048 | 0.368 ± 0.056 | 0.594 ± 0.008 | 0.769 ± 0.004 | 0.98 ± 0.003 |
| CB2 | 0.92 ± 0.045 | 0.466 ± 0.149 | 0.609 ± 0.206 | 0.636 ± 0.007 | 1.122 ± 0.03 | 1.435 ± 0.033 | 0.793 ± 0.014 | 1.181 ± 0.014 | 1.546 ± 0.021 | 0.944 ± 0.013 | 0.286 ± 0.042 | 0.35 ± 0.052 | 0.582 ± 0.011 | 0.944 ± 0.008 | 0.739 ± 0.006 |
| MAGL | 0.859 ± 0.009 | 0.662 ± 0.022 | 0.825 ± 0.024 | 0.682 ± 0.011 | 0.949 ± 0.012 | 1.189 ± 0.017 | 0.669 ± 0.015 | 1.184 ± 0.013 | 1.436 ± 0.018 | 0.968 ± 0.01 | 0.278 ± 0.045 | 0.338 ± 0.055 | 0.61 ± 0.019 | 0.84 ± 0.013 | 1.069 ± 0.014 |
| AchE | 0.798 ± 0.024 | 0.775 ± 0.038 | 0.962 ± 0.041 | 0.642 ± 0.029 | 0.915 ± 0.011 | 1.111 ± 0.013 | 0.726 ± 0.018 | 1.037 ± 0.035 | 1.25 ± 0.042 | 0.964 ± 0.009 | 0.298 ± 0.035 | 0.388 ± 0.038 | 0.739 ± 0.008 | 0.727 ± 0.012 | 1.126 ± 0.023 |
| D_1_ | 0.797 ± 0.014 | 0.618 ± 0.007 | 0.765 ± 0.006 | 0.694 ± 0.008 | 0.729 ± 0.005 | 0.901 ± 0.009 | 0.656 ± 0.011 | 0.976 ± 0.016 | 1.174 ± 0.016 | 0.942 ± 0.019 | 0.298 ± 0.039 | 0.366 ± 0.041 | 0.72 ± 0.009 | 0.755 ± 0.013 | 0.956 ± 0.012 |
| D_2_ | 0.816 ± 0.01 | 0.514 ± 0.007 | 0.626 ± 0.009 | 0.569 ± 0.005 | 0.796 ± 0.006 | 0.983 ± 0.008 | 0.46 ± 0.016 | 0.951 ± 0.006 | 1.131 ± 0.007 | 0.934 ± 0.022 | 0.282 ± 0.032 | 0.354 ± 0.036 | 0.57 ± 0.01 | 0.672 ± 0.006 | 0.905 ± 0.009 |
| α_2_ | 0.837 ± 0.009 | 0.504 ± 0.008 | 0.61 ± 0.01 | 0.587 ± 0.005 | 0.8 ± 0.008 | 1.003 ± 0.01 | 0.529 ± 0.012 | 1.022 ± 0.003 | 1.201 ± 0.001 | 0.976 ± 0.016 | 0.254 ± 0.028 | 0.32 ± 0.032 | 0.669 ± 0.011 | 1.023 ± 0.042 | 1.433 ± 0.051 |
| M_2_ | 0.787 ± 0.008 | 0.471 ± 0.005 | 0.584 ± 0.005 | 0.578 ± 0.005 | 0.741 ± 0.003 | 0.946 ± 0.005 | 0.416 ± 0.009 | 0.864 ± 0.004 | 1.078 ± 0.005 | 0.964 ± 0.011 | 0.312 ± 0.031 | 0.392 ± 0.037 | 0.701 ± 0.012 | 0.983 ± 0.019 | 1.23 ± 0.016 |
| NMDA | 0.82 ± 0.004 | 0.79 ± 0.009 | 0.955 ± 0.009 | 0.681 ± 0.01 | 0.961 ± 0.01 | 1.183 ± 0.007 | 0.611 ± 0.015 | 1.255 ± 0.018 | 1.494 ± 0.017 | 0.962 ± 0.013 | 0.28 ± 0.041 | 0.344 ± 0.047 | 0.608 ± 0.01 | 0.931 ± 0.009 | 1.159 ± 0.006 |
| GRK2 | 0.835 ± 0.008 | 0.638 ± 0.01 | 0.811 ± 0.01 | 0.61 ± 0.004 | 1.005 ± 0.009 | 1.344 ± 0.026 | 0.546 ± 0.004 | 1.264 ± 0.012 | 1.648 ± 0.029 | 0.938 ± 0.027 | 0.32 ± 0.066 | 0.398 ± 0.09 | 0.61 ± 0.019 | 0.84 ± 0.013 | 1.069 ± 0.014 |

**Table S5**. Summarizes statistical characteristics of the validation set multitasking and single-task regression models developed using scafold split

| **Assay** | **DNN** | | | **RF** | | | **LightGBM** | | | **MT-DNN** | | | **SVM** | | |
| --- | --- | --- | --- | --- | --- | --- | --- | --- | --- | --- | --- | --- | --- | --- | --- |
|  | ***r*** | **MAE** | **RMSE** | ***r*** | **MAE** | **RMSE** | ***r*** | **MAE** | **RMSE** | ***r*** | **MAE** | **RMSE** | ***r*** | **MAE** | **RMSE** |
| CB1 | 0.84 ± 0.026 | 0.628 ± 0.042 | 0.813 ± 0.057 | 0.128 ± 0.038 | 1.242 ± 0.035 | 1.656 ± 0.056 | 0.104 ± 0.019 | 1.253 ± 0.035 | 1.657 ± 0.056 | 0.764 ± 0.059 | 0.59 ± 0.033 | 0.72 ± 0.046 | 0.23 ± 0.036 | 1.041 ± 0.039 | 1.267 ± 0.04 |
| CB2 | 0.376 ± 0.035 | 1.131 ± 0.06 | 1.39 ± 0.079 | 0.266 ± 0.048 | 1.23 ± 0.032 | 1.636 ± 0.057 | -0.097 ± 0.05 | 1.242 ± 0.035 | 1.682 ± 0.055 | 0.76 ± 0.015 | 0.598 ± 0.039 | 0.728 ± 0.043 | -0.029 ± 0.088 | 1.247 ± 0.073 | 1.494 ± 0.065 |
| MAGL | 0.31 ± 0.032 | 1.2 ± 0.067 | 1.476 ± 0.08 | 0.114 ± 0.022 | 1.255 ± 0.035 | 1.641 ± 0.056 | 0.054 ± 0.021 | 1.298 ± 0.041 | 1.675 ± 0.064 | 0.796 ± 0.115 | 0.634 ± 0.112 | 0.768 ± 0.134 | 0.317 ± 0.045 | 1.021 ± 0.043 | 1.244 ± 0.042 |
| AchE | 0.07 ± 0.044 | 1.157 ± 0.058 | 1.444 ± 0.081 | 0.253 ± 0.039 | 1.256 ± 0.04 | 1.634 ± 0.063 | 0.251 ± 0.027 | 1.292 ± 0.041 | 1.668 ± 0.065 | 0.788 ± 0.032 | 0.672 ± 0.054 | 0.846 ± 0.074 | 0.22 ± 0.045 | 1.206 ± 0.054 | 1.525 ± 0.043 |
| D_1_ | 0.005 ± 0.029 | 1.127 ± 0.052 | 1.374 ± 0.07 | 0.079 ± 0.031 | 1.356 ± 0.039 | 1.753 ± 0.065 | 0.014 ± 0.012 | 1.477 ± 0.042 | 1.851 ± 0.069 | 0.746 ± 0.122 | 0.56 ± 0.064 | 0.692 ± 0.075 | 0.365 ± 0.031 | 1.05 ± 0.039 | 1.254 ± 0.045 |
| D_2_ | 0.044 ± 0.041 | 1.199 ± 0.059 | 1.473 ± 0.069 | -0.121 ± 0.031 | 1.595 ± 0.044 | 1.965 ± 0.07 | 0.006 ± 0.046 | 1.627 ± 0.041 | 1.995 ± 0.07 | 0.786 ± 0.011 | 0.502 ± 0.023 | 0.624 ± 0.026 | 0.284 ± 0.03 | 1.037 ± 0.045 | 1.246 ± 0.046 |
| α_2_ | 0.085 ± 0.031 | 1.129 ± 0.057 | 1.397 ± 0.07 | 0.073 ± 0.052 | 1.374 ± 0.04 | 1.768 ± 0.066 | 0.142 ± 0.032 | 1.558 ± 0.042 | 1.925 ± 0.07 | 0.766 ± 0.103 | 0.562 ± 0.09 | 0.706 ± 0.136 | 0.232 ± 0.03 | 1.051 ± 0.052 | 1.33 ± 0.056 |
| M_2_ | 0.139 ± 0.022 | 1.159 ± 0.036 | 1.413 ± 0.056 | 0.239 ± 0.033 | 1.389 ± 0.044 | 1.777 ± 0.07 | 0.025 ± 0.04 | 1.472 ± 0.043 | 1.847 ± 0.07 | 0.804 ± 0.031 | 0.716 ± 0.064 | 0.886 ± 0.083 | 0.137 ± 0.024 | 1.128 ± 0.058 | 1.351 ± 0.057 |
| NMDA | 0.081 ± 0.049 | 1.212 ± 0.057 | 1.496 ± 0.073 | -0.036 ± 0.031 | 1.415 ± 0.045 | 1.805 ± 0.069 | 0.015 ± 0.023 | 1.56 ± 0.042 | 1.927 ± 0.07 | 0.656 ± 0.1 | 0.79 ± 0.084 | 0.98 ± 0.135 | 0.774 ± 0.025 | 0.666 ± 0.02 | 0.873 ± 0.035 |
| GRK2 | 0.923 ± 0.008 | 0.437 ± 0.022 | 0.578 ± 0.036 | 0.628 ± 0.031 | 1.008 ± 0.025 | 1.364 ± 0.062 | 0.759 ± 0.022 | 1.269 ± 0.038 | 1.65 ± 0.061 | 0.602 ± 0.265 | 0.818 ± 0.335 | 1.022 ± 0.477 | 0.268 ± 0.091 | 1.022 ± 0.052 | 1.293 ± 0.046 |

**Table S5**. Summarizes statistical characteristics of the train set multitasking and single-task regression models developed using scafold split

| **Assay** | **DNN** | | | **RF** | | | **LightGBM** | | | **MT-DNN** | | | **SVM** | | |
| --- | --- | --- | --- | --- | --- | --- | --- | --- | --- | --- | --- | --- | --- | --- | --- |
|  | r | MAE | RMSE | r | MAE | RMSE | r | MAE | RMSE | r | MAE | RMSE | r | MAE | RMSE |
| CB1 | 0.831 ± 0.003 | 0.638 ± 0.008 | 0.829 ± 0.012 | 0.125 ± 0.009 | 1.241 ± 0.01 | 1.66 ± 0.023 | 0.101 ± 0.004 | 1.25 ± 0.009 | 1.661 ± 0.023 | 0.938 ± 0.013 | 0.334 ± 0.028 | 0.406 ± 0.035 | 1.046 ± 0.006 | 1.046 ± 0.006 | 1.268 ± 0.006 |
| CB2 | 0.347 ± 0.008 | 1.155 ± 0.01 | 1.42 ± 0.012 | 0.251 ± 0.011 | 1.226 ± 0.009 | 1.637 ± 0.023 | -0.075 ± 0.011 | 1.241 ± 0.01 | 1.687 ± 0.023 | 0.938 ± 0.01 | 0.314 ± 0.023 | 0.386 ± 0.027 | 1.263 ± 0.014 | 1.263 ± 0.014 | 1.526 ± 0.011 |
| MAGL | 0.284 ± 0.004 | 1.203 ± 0.011 | 1.484 ± 0.015 | 0.136 ± 0.006 | 1.251 ± 0.008 | 1.641 ± 0.023 | 0.073 ± 0.004 | 1.289 ± 0.01 | 1.673 ± 0.024 | 0.964 ± 0.008 | 0.296 ± 0.031 | 0.364 ± 0.045 | 1.023 ± 0.01 | 1.023 ± 0.01 | 1.239 ± 0.008 |
| AchE | 0.107 ± 0.011 | 1.149 ± 0.013 | 1.434 ± 0.019 | 0.236 ± 0.009 | 1.253 ± 0.01 | 1.637 ± 0.024 | 0.24 ± 0.007 | 1.285 ± 0.01 | 1.668 ± 0.024 | 0.964 ± 0.005 | 0.312 ± 0.017 | 0.402 ± 0.021 | 1.199 ± 0.016 | 1.199 ± 0.016 | 1.512 ± 0.018 |
| D_1_ | 0.007 ± 0.009 | 1.142 ± 0.011 | 1.397 ± 0.017 | 0.042 ± 0.005 | 1.347 ± 0.01 | 1.752 ± 0.024 | -0.019 ± 0.008 | 1.461 ± 0.011 | 1.846 ± 0.023 | 0.94 ± 0.009 | 0.294 ± 0.013 | 0.362 ± 0.019 | 1.042 ± 0.007 | 1.042 ± 0.007 | 1.256 ± 0.008 |
| D_2_ | 0.018 ± 0.01 | 1.22 ± 0.015 | 1.502 ± 0.016 | -0.084 ± 0.008 | 1.574 ± 0.011 | 1.956 ± 0.023 | 0.019 ± 0.007 | 1.607 ± 0.011 | 1.987 ± 0.023 | 0.928 ± 0.008 | 0.298 ± 0.013 | 0.368 ± 0.013 | 1.036 ± 0.01 | 1.036 ± 0.01 | 1.245 ± 0.008 |
| α_2_ | 0.115 ± 0.008 | 1.129 ± 0.006 | 1.391 ± 0.008 | 0.072 ± 0.006 | 1.364 ± 0.011 | 1.766 ± 0.023 | 0.114 ± 0.007 | 1.539 ± 0.011 | 1.918 ± 0.023 | 0.974 ± 0.005 | 0.276 ± 0.027 | 0.346 ± 0.032 | 1.119 ± 0.009 | 1.119 ± 0.009 | 1.41 ± 0.007 |
| M_2_ | 0.121 ± 0.007 | 1.173 ± 0.011 | 1.423 ± 0.015 | 0.234 ± 0.006 | 1.374 ± 0.011 | 1.772 ± 0.024 | 0.048 ± 0.002 | 1.456 ± 0.011 | 1.841 ± 0.023 | 0.958 ± 0.01 | 0.332 ± 0.034 | 0.418 ± 0.044 | 1.163 ± 0.01 | 1.163 ± 0.01 | 1.391 ± 0.009 |
| NMDA | 0.082 ± 0.009 | 1.228 ± 0.011 | 1.519 ± 0.015 | -0.009 ± 0.006 | 1.402 ± 0.011 | 1.798 ± 0.023 | 0.026 ± 0.014 | 1.542 ± 0.011 | 1.92 ± 0.023 | 0.954 ± 0.005 | 0.308 ± 0.016 | 0.384 ± 0.02 | 0.656 ± 0.002 | 0.656 ± 0.002 | 0.86 ± 0.003 |
| GRK2 | 0.926 ± 0.002 | 0.422 ± 0.004 | 0.553 ± 0.009 | 0.612 ± 0.005 | 1.005 ± 0.009 | 1.364 ± 0.025 | 0.763 ± 0.004 | 1.264 ± 0.009 | 1.652 ± 0.024 | 0.914 ± 0.037 | 0.364 ± 0.056 | 0.456 ± 0.068 | 1.038 ± 0.008 | 1.038 ± 0.008 | 1.296 ± 0.007 |

**Table S6.** Hyperparameter Configuration for FNN, Random Forest, and LightGBM Models in a Single-Task Approach

| Model | Hyperparameter | Values |
| --- | --- | --- |
| Feedforward Neural Network  (FNN) | hidden_layers | 2 |
|  | neurons_per_layer | Input: 50, hidden_layers: 50 |
|  | activation_function | relu, selu, selu, linear |
|  | learning_rate | 0.01 |
|  | optimizer | RMSprop |
|  | batch_size | 48 |
|  | epochs | 1000 |
| Random Forest  (RF) | max_depth | 9 |
|  | max_features | sqrt |
|  | min_samples_leaf | 19 |
|  | min_samples_split | 8 |
|  | n_estimators | 68 |
| Light Gradient-Boosting Machine  (LGBM) | learning_rate | 0.07756 |
|  | max_depth | 9 |
|  | n_estimators | 12 |
|  | num_leaves | 49 |
|  | scale_pos_weight | 189 |
|  | subsample | 0.6721 |
| Support Vector Machines  (SVM) | C | 0.01 |
|  | Kernel | Linear e rbf |
|  | Gamma | 0.1 |
|  |  |  |

The hyperparameter values presented in this table were applied in the random split method

**Table S7.** Distribution of data points across the training, validation, and test sets for each task. Each set corresponds to a specific phase of model development and evaluation.

| Tasks | Train (80%) | Validation (10%) | Test (10%) |
| --- | --- | --- | --- |
| Number of Data Points | | | |
| CB1 | 2063 | 258 | 258 |
| CB2 | 2736 | 342 | 342 |
| MAGL | 529 | 66 | 66 |
| AchE | 3754 | 469 | 469 |
| D1 | 784 | 98 | 98 |
| D2 | 4906 | 613 | 613 |
| α2 | 361 | 45 | 45 |
| M2 | 947 | 118 | 118 |
| NMDA | 1113 | 139 | 139 |
| GRK2 | 238 | 30 | 30 |
| **Multitasking approach** | **17,430** | **2,179** | **2,178** |

ach


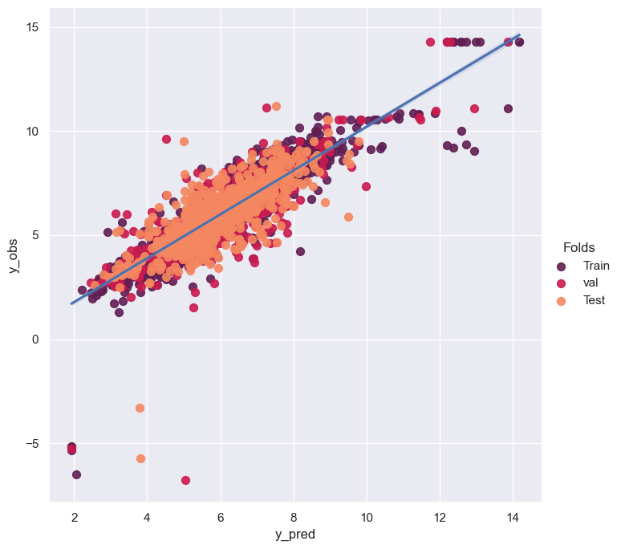

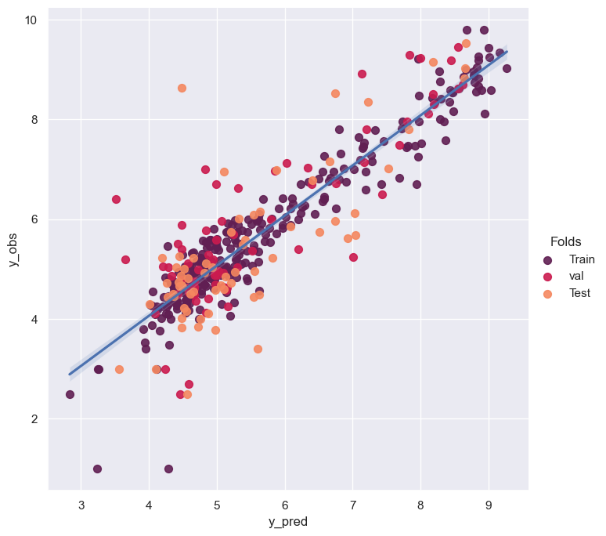


α 2


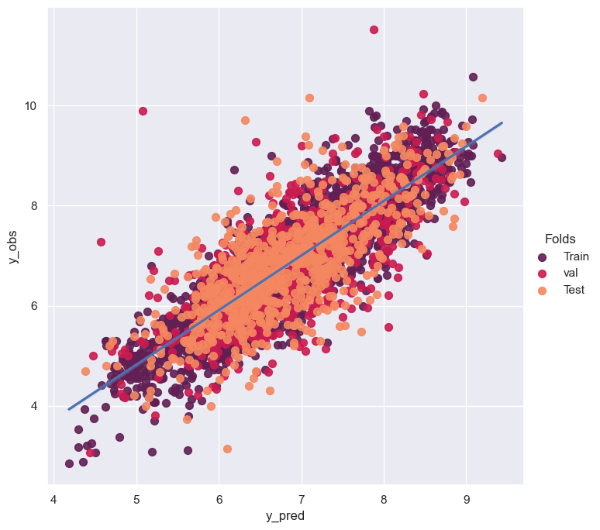


CB1


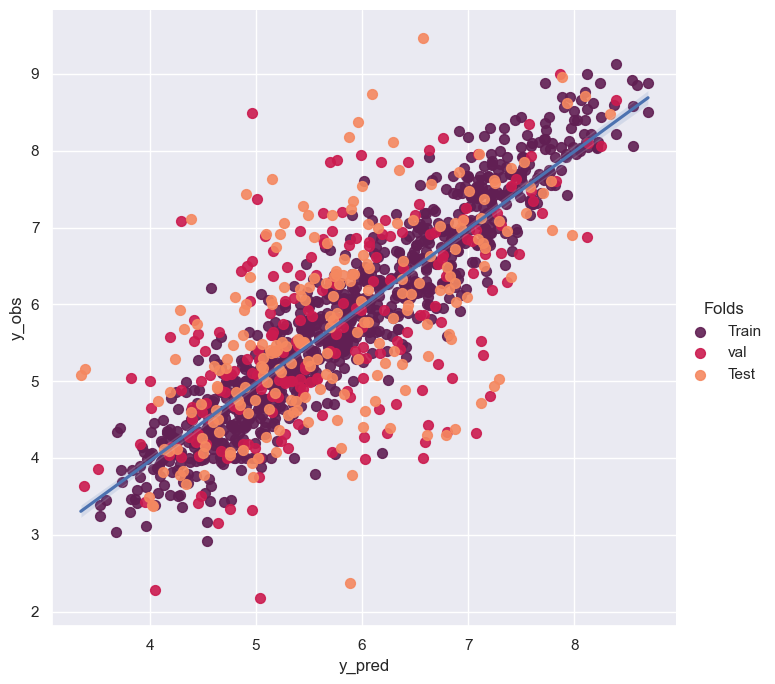


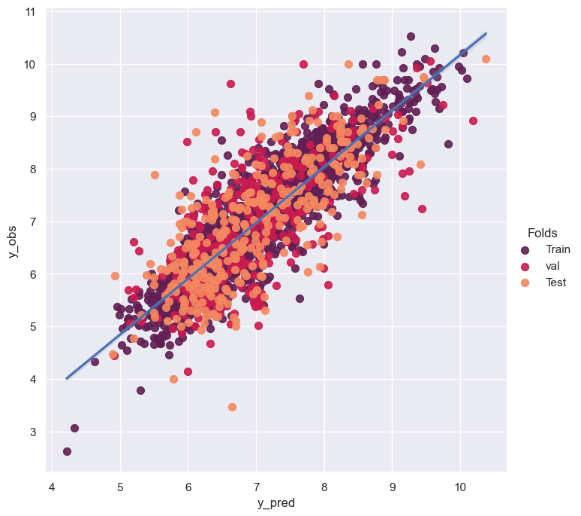


CB2


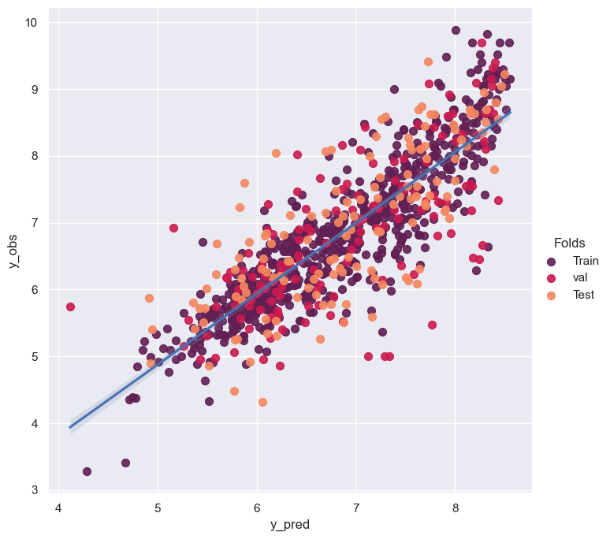


D1


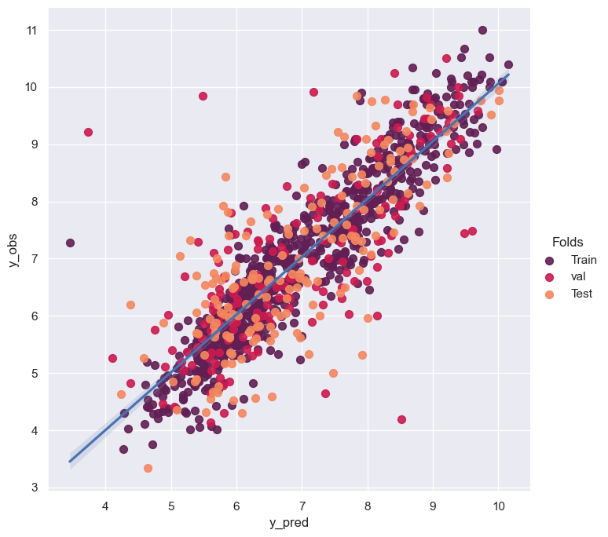


D2


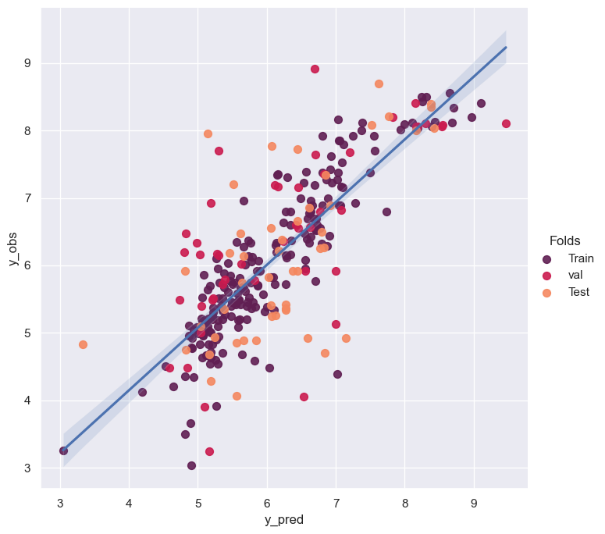


G2


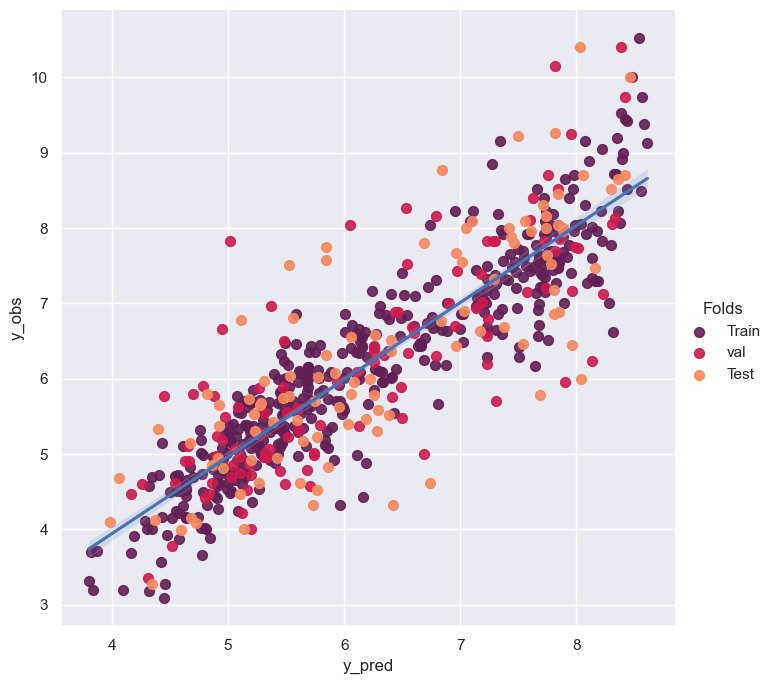


NMDA

MAGL


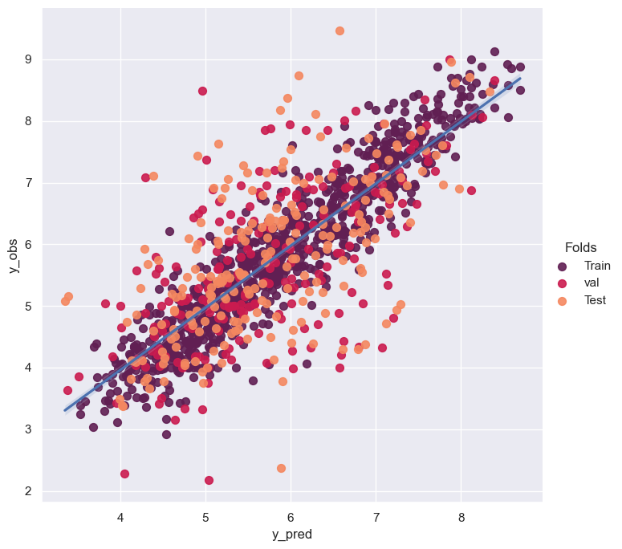

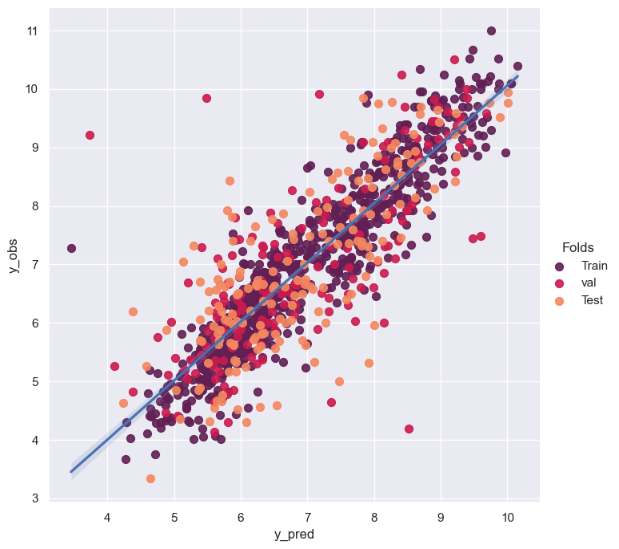


M2

**Fig. S3.** Scatter plot illustrating the correlation between predicted and experimental pIC₅₀ values for the selected tasks MIE1, KE1, and KE2. CB1: cannabinoid receptor 1; CB2: cannabinoid receptor 2; MAGL: Monoacylglycerol lipase; AchE: acetylcholinesterase; D_1_: dopamine 1 receptor; D_2_: dopamine 2 receptor; α_2_: adrenergic receptor alpha-2; M_2_: muscarinic acetylcholine receptor M2; NMDA: N-methyl-D-aspartate glutamate receptor; GRK2: G-protein coupled receptor kinase 2.


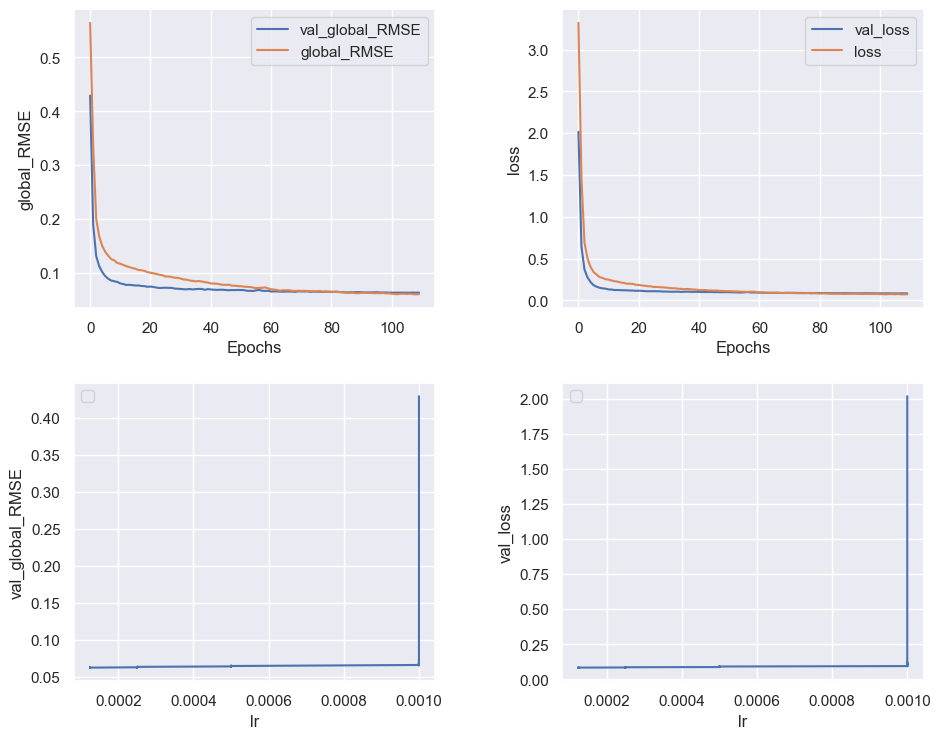


**(b)**


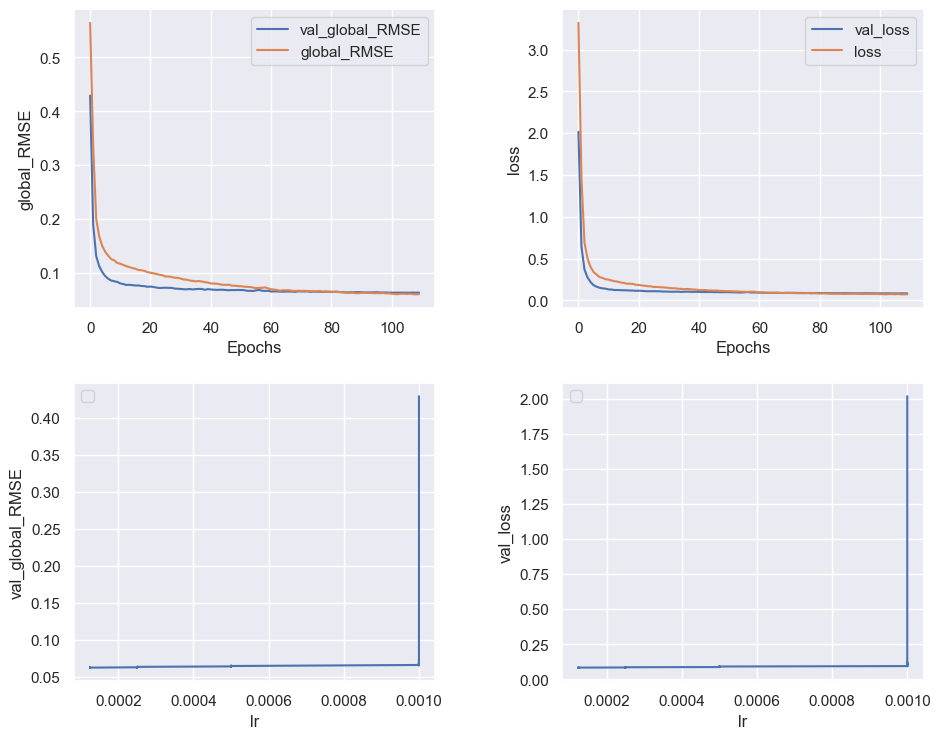


**(a)**


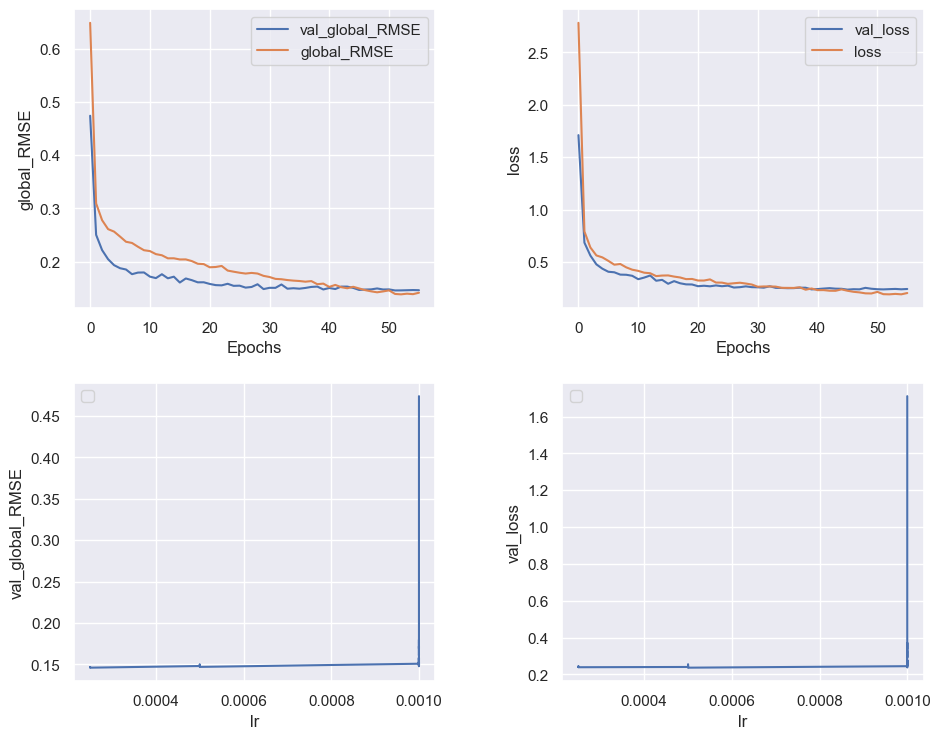


**(d)**


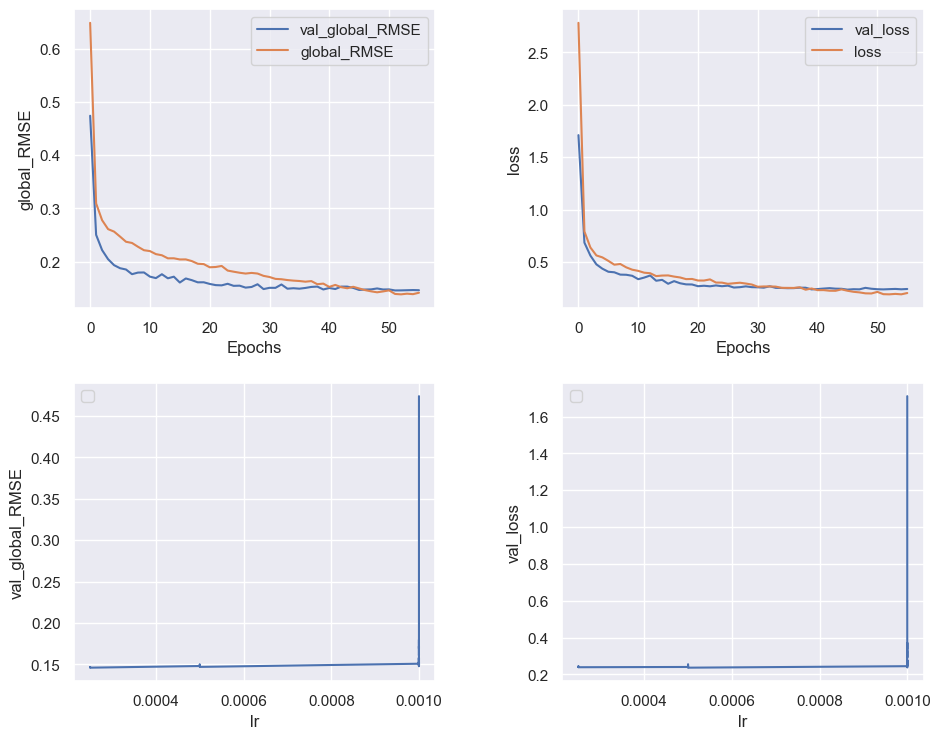


**(c)**

**Fig. S3** Comparative analysis of learning curves for a generalized Multi-Task model (10 tasks) versus a specific Single-Task model (using Acetylcholinesterase as an example). **(a, c)** Single-Task Model (Acetylcholinesterase): Progression of the Root Mean Square Error (RMSE) and loss function on the training and validation sets. **(b, d)** Multi-Task Model: Progression of the global RMSE and global loss across all tasks. The Multi-Task model demonstrates robust convergence and lower final validation error, indicating that the shared representations learned from multiple related tasks enhance predictive performance and generalization compared to training on a single task in isolation.


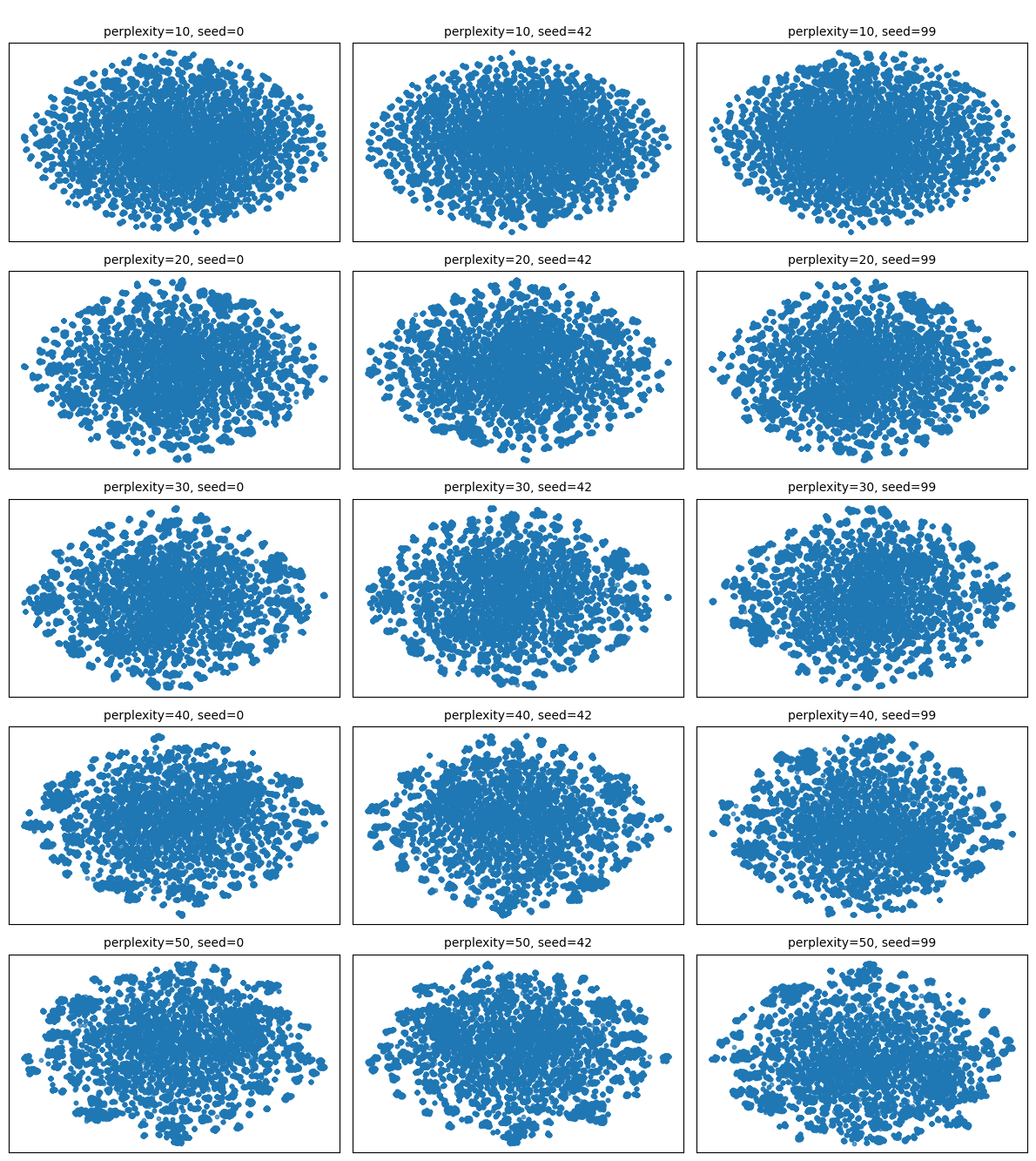


**(a)**


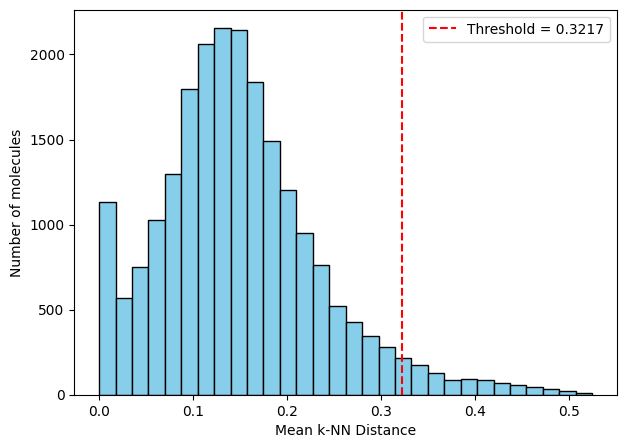


**(b)**

**Fig. S4** t-SNE parameter optimization and Applicability Domain definition. **(a)** t-SNE embeddings across perplexity values (10–50) and random seeds show consistent chemical space structure. **(b)** k-NN distance distribution defines AD threshold (0.3217), separating reliable prediction regions (inside AD) from extrapolation zones (outside AD).


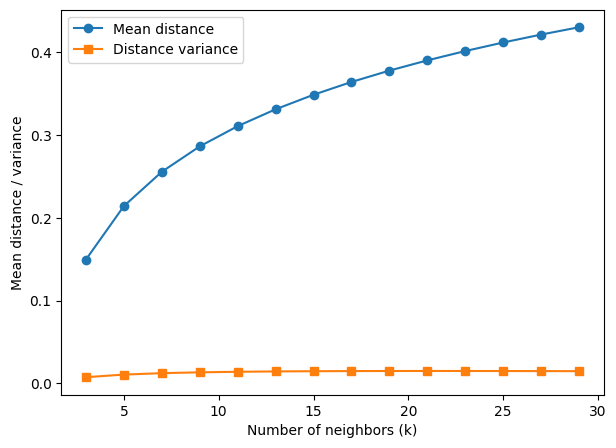


**Fig. S5.** Analysis of local neighborhood distance statistics as a function of the number of neighbors, k. The plot shows the mean distance to the k-th nearest neighbor (blue line), the variance of these distances (orange line), and their ratio (Mean/Variance, green line). The mean distance increases with k as more distant neighbors are included. The variance and the mean-to-variance ratio are often used to assess the intrinsic dimensionality and local data density; an optimal k for neighborhood-based algorithms often corresponds to an extremum (peak or trough) in the ratio curve, indicating a scale where the local manifold structure is most stable.
